# DNA2 Nuclease Inhibition Confers Synthetic Lethality in Cancers with Mutant p53 and Synergizes with PARP Inhibitors

**DOI:** 10.1158/2767-9764.CRC-23-0166

**Published:** 2023-10-16

**Authors:** Helena Folly-Kossi, Joshua D. Graves, Lidija A. Wilhelms Garan, Fang-Tsyr Lin, Weei-Chin Lin

**Affiliations:** 1 Section of Hematology/Oncology, Department of Medicine, Baylor College of Medicine, Houston, Texas.; 2 Integrative Molecular and Biomedical Sciences Graduate Program, Baylor College of Medicine, Houston, Texas.; 3 Dan L. Duncan Comprehensive Cancer Center, Baylor College of Medicine, Houston, Texas.; 4 Department of Molecular and Cellular Biology, Baylor College of Medicine, Houston, Texas.

## Abstract

The tumor suppressor p53 promotes tumor-suppressive activities including cell-cycle inhibition, apoptosis, senescence, autophagy, and DNA repair. However, somatic mutations in the *TP53* gene are one of the most common alterations in human cancers. We previously showed that mutant p53 (mutp53) can bind TopBP1, an ATR activator, to attenuate its ATR-activating function. A partially defective ATR function caused by mutp53 makes cancer cells more vulnerable to inhibitors of other TopBP1-independent ATR activators, such as DNA2. DNA2 plays a role in homologous recombination (HR) repair by resecting DNA ends in double-strand breaks and preparing them for invasion of homologous duplex. Here we identify a new DNA2 inhibitor, namely d16, and show that d16 exhibits anticancer activities and overcomes chemotherapy resistance in mutp53-bearing cancers. Similar to DNA2 depletion, d16 treatment results in cell-cycle arrest mainly at S-phase. Moreover, reexpression of mutp53 in a p53-null cancer cell line makes cells more vulnerable to d16-mediated inhibition of ATR activity. As d16 also inhibits HR, a combination of d16 and PARP inhibitors displays synergistic induction of cell death. DNA2 is often overexpressed in cancer, particularly in cancer cells harboring mutp53. Overexpression of DNA2 is associated with poor outcome in ovarian cancer. Overall, our results provide a rationale to target DNA2 as a new synthetic lethality approach in mutp53-bearing cancers, and further extend the benefit of PARP inhibitors beyond BRCA-mutated cancers.

**Significance:**

This study identifies a new DNA2 inhibitor as a synthetic lethal targeted therapy for mutp53-harboring cancers, and provides a new therapeutic strategy by combining DNA2 inhibitors with PARP inhibitors for these cancers.

## Introduction

Normal cellular growth is regulated by replication checkpoints. Therefore, to maintain genomic integrity, it is crucial to ensure that DNA damage, such as DNA double-strand breaks (DSB), are repaired accurately. One of the major cellular stress sensors triggered by DSBs involves transcription factor p53. The tumor suppressor p53 promotes tumor-suppressive activities including cell-cycle inhibition, apoptosis, senescence, autophagy, and DNA repair, etc. By stopping aberrant DNA replication characterized by oncogenic pressure, p53 function constitutes one of the most powerful shields against tumor growth (1). In fact, The Cancer Genome Atlas (TCGA) study, including the Pan-Cancer project, identifies *TP53* gene as the most significantly mutated gene in human cancers, resulting in perturbation of replication checkpoint function (2–5). Hence, to unravel new approaches to halt cancer progression and improve the efficacy of cancer therapy, it is essential to understand the contribution of *TP53* mutations in response to DNA damage. One of the mechanisms caused by *TP53* mutations (mutp53) involves the checkpoint activator TopBP1 (topoisomerase IIβ-binding protein), a critical molecule capable of facilitating the complex formation between several hotspot mutp53 proteins and either (nuclear factor Y) NF-Y or p63/p73 (6). As a checkpoint facilitator, upon DNA replication stress, TopBP1 binds directly to stalled forks and recruits the Rad9–Hus1–Rad1 (9-1-1) complex (7) to activate ATR, one of the major key regulators in checkpoint responses to DNA damage (8). ATR activation is mediated through a conserved ATR-activating domain located between the sixth and seventh BRCT domains of TopBP1 (8). Previously, our lab reported that several hotspot p53 mutants are capable of binding TopBP1 and interfering with its ATR-activating function by inducing TopBP1 oligomerization independently of Akt (9). As a result, the ATR/Chk1 S-phase checkpoint response is decreased upon replication stress in many mutp53-harboring cancer cells (9). It is noteworthy that ATR can also be activated by DNA2 (DNA nuclease/helicase 2; refs. 10–12) or ETAA1 (Ewing tumor-associated antigen 1; refs. 13–15) independently of TopBP1. Although TopBP1-mediated Chk1 activation is inhibited by mutp53, ATR function is only partially attenuated in cancer cells harboring mutp53, probably due to intact DNA2 and ETAA1 pathways. ATR is indispensable for cell survival because it is required to regulate replication origin firing, stabilize stalled replication forks, and promote DNA repair. In fact, many studies have reported that a complete loss of function of ATR/Chk1 leads to cell death (16–18). Loss-of-function mutations in the ATR pathway are rarely observed, suggesting that this pathway is required for cell survival (19). Therefore, targeting DNA2 in mutp53-bearing cancers may severely cripple ATR function, thereby inhibiting cell survival and enhancing chemosensitivity.

In addition to the functions mentioned above, DNA2 has been shown to promote homologous recombination (HR) repair by facilitating the recruitment of HR repair factors, such as Rad51, to DSBs (20). DNA2 possesses the nuclease activity to resect the DNA ends in DSBs and prepare them for invasion of homologous duplex. Therefore, targeting DNA2 may impair the HR pathway, providing a rationale to exploit the synthetic lethal strategy by combining DNA2 inhibitors with PARP inhibitors (PARPi). This would greatly extend the benefit of PARPi beyond BRCA-mutated cancers, which currently account for less than 15% of breast and ovarian cancers. Previously, two DNA2 inhibitors, NSC-15765 (also known as C5; ref. 21) and NSC-105808 (22), have been identified. However, there are no data to support their *in vivo* antitumor activities and their anticancer activities remain to be optimized.

Here, we identify a new DNA2 inhibitor, namely d16, and demonstrate the synthetic lethal potential of DNA2 inhibitors for treating mutant p53-harboring cancers. Our data show that d16 exerts *in vivo* anticancer activity in an ovarian cancer xenograft model. Moreover, d16 can inhibit HR and synergize with PARPis in several BRCA-proficient ovarian and breast cancer cells, providing a new therapeutic strategy for the treatment of *TP53*-mutated, *BRCA1/2*-WT (wild-type), breast and ovarian cancers.

## Materials and Methods

### Molecular Docking

Crystal structure of Dna2 nuclease-helicase was downloaded from the Protein Data Bank (accession no. 5EAW). Molecular docking study was performed using 1-Click Docking of Mcule software (https://mcule.com/apps/1-click-docking/).

### Compounds

DNA2 inhibitor C5 (NSC-15765) was obtained from NCI Developmental Therapeutics Program (DTP, RRID:SCR_003057). C5 analogs were purchased from MolPort, Inc. or ChemBridge Corporation. Compound d16 was resynthesized in ChemBridge Corporation.

### Cell Culture and Transfection

A2780cis cell line (ECACC 93112517) was purchased from PHE Culture collections through Sigma. Other human cell lines were purchased from ATCC. MDA-MB-468 and BT549 human breast cancer cells, H1299 human lung cancer cells, C33A human cervical cancer cells (BCRJ catalog no. 0348, RRID:CVCL_1094) and MDAH-2774 ovarian cancer cells (BCRJ catalog no. 0163, RRID:CVCL_0420) were grown in DMEM, supplemented with 10% FBS, penicillin (50 IU/mL), and streptomycin (50 µg/mL) in a humidified incubator with 5% CO_2_ at 37°C. All cell lines were confirmed negative for *Mycoplasma* contamination using PlasmoTest *Mycoplasma* Detection Kit (InvivoGen, catalog no. rep-pt1) according to manufacturer's instruction at the time of stock vial preparation. Cells were authenticated by ATCC short tandem repeat profiling and routine examination of morphology and consistent *in vitro* growth properties. Cell lines were grown for no more than 15 passages after thawing. For transient transfection, WT p53 plasmid and single hotspot mutant p53 plasmid constructs including V143A, R175H, R248W, R249S, and R273H (6) were transfected into H1299 cells with polyethylenimine (PEI). Cells were treated or harvested 36 to 48 hours after transfection for analysis.

### RNA Extraction and qRT-PCR

RNA was extracted from H1299 lung cancer cells using TRIzol reagent (Invitrogen), and quantitative PCR (QPCR) was performed in triplicates on MX3005P thermal cycler using SYBR Q-PCR Master Mix (GenDEPOT). The primer pairs used for DNA2 amplification are 5′-ATTTCTGGCACCAGCATAGC-3′ and5′-GCTGGATAGCTGGATGAACC-3′, and the primer pairs used for GAPDH amplification are 5′-ATTGGGCGCCTGGTCACCAGGGCTG-3′ and 5′-AAATGAGCCCCAGCCTTCTCCATG-3′. The DNA2 mRNA levels were analyzed with MxPro 4.0 QPCR software (Stratagene; RRID:SCR_016375) and were normalized relative to GAPDH.

### Antibodies and Western Blotting and Immunoprecipitation

Cells were lysed in 1% SDS buffer. After boiling, the lysates were sonicated and subjected to centrifugation at 20,000 × *g* for 20 minutes. Supernatant were boiled in SDS loading buffer, separated by SDS-PAGE and electrotransferred to the polyvinylidene fluoride membrane (Millipore). After blocking with 5% milk, immunoblotting was performed with the appropriate antibodies. Antibodies specific to p53 (Santa Cruz Biotechnology catalog no. sc-6243, RRID:AB_653753), Chk1 (G-4), GAPDH (6C5), and Actin (C-2) were from Santa Cruz Biotechnology. Antibodies specific to phospho-Chk1 (S345) and RAD51 (Cell Signaling Technology catalog no. 8875, RRID:AB_2721109) were from Cell Signaling Technology. Antibody specific to DNA2 (Thermo Fisher Scientific catalog no. PA5-68167, RRID:AB_2691658) was from Thermo Fisher Scientific. Antibody specific to PARP (Asp 214) was from BD Biosciences.

### IHC Analysis

Xenografts tissues were harvested, and fixed in 10% neutral buffered formalin (∼4% formaldehyde) for 24–72 hours. Tissues were then placed in 70% ethanol at 4°C. IHC was performed by staining tissues using hematoxylin and eosin (H&E), cleaved PARP1 and Ki67 antibodies under the care of the pathology core and lab of Baylor College of Medicine (BCM) using standard operating protocols.

### Combination Index Analysis

To determine and quantify a dose-effect relationship between DNA2 inhibitor d16 and PARPi [rucaparib (RU) or talazoparib], different doses of d16 or PARPi, or combinations of d16 and PARPi with a fixed constant ratio were added to BT549, MDA-MB-468, MDAH-2774, and C33A cells, respectively. The combinational effect was evaluated using the combination index (CI) method as described previously (23, 24). The mode of interaction (synergy, antagonism, or additivity) was determined using CompuSyn software program (CompuSyn, Inc.). CI is a quantitative measurement of the degree of drug interaction (CI = 1, additive effects), (CI < 1, synergy), (CI > 1, antagonism).

### Data Mining

The UALCAN platform (25) was used to analyze TCGA database. UALCAN database was employed to analyze the mRNA expression of DNA2 in normal, *TP53* mutant and WT *TP53* tissues in all types of cancer from TCGA datasets. CSIOVDB (26), a microarray database of ovarian cancer data, was used to analyze the DNA2 mRNA expression and its relationship with clinicopathologic parameters (cancer state, histologic subtypes, International Federation of Gynecology and Obstetrics (FIGO) grade, FIGO stage, clinical response, and molecular subtypes). CSIOVDB database was also used to generate Kaplan–Meier survival plots comparing the lower and upper quartiles of DNA2 gene expression in epithelial ovarian carcinoma (EOC) and in stem-like-B (Stem-B) molecular subtype EOC whole population using the median to distinguish between high and low levels of DNA2 mRNA expression. Box plots of DNA2 mRNA expression in chemotherapy responder or nonresponder patients of serous ovarian cancer (defined by relapse-free survival at 6 months) were generated from TCGA dataset using ROC plotter platform (27).

### Cell Proliferation, Apoptosis, and Clonogenic Survival Assay

Cells were seeded on 96-well plates at 2,000 cells per well, and were treated with various concentrations of DNA2 inhibitor d16 for 72 hours. Next, 20 µL of MTT reagent (thiazolyl blue tetrazolium bromide, 5 mg/mL; Sigma) was added to each well, followed by incubation at 37°C for 1 hour. The medium and reagent were washed away and 150 µL of DMSO was added to each well. After incubation at room temperature for 15 minutes, absorbance was read at 490 nm on a plate reader (BioTek Synergy HT). Each experiment was performed at least in triplicates. Apoptosis was determined by the Caspase-Glo 3/7 activity assay (Promega), which measures the caspase-3/7 activity by cleavage of the luminogenic substrate containing the DEVD sequence, and was normalized to protein concentrations. Clonogenic survival assay was performed as described previously (6). Briefly, cells were seeded on 6-well plates at 300 cells per well. After 24 hours, cells were treated with the indicated drugs for 24 hours, washed twice with PBS and then incubated in fresh culture medium for 8–12 days. The colonies were fixed with 4% formaldehyde, and then stained with 0.5% crystal violet in 25% methanol solution.

### Flow Cytometry and Bromodeoxyuridine Incorporation Assay

Propidium iodide (PI) staining/flow cytometry and bromodeoxyuridine (BrdU) incorporation assay were performed as describe previously (28). The data were analyzed using FlowJo software (RRID:SCR_008520).

### Preparation of DNA Substrate for Nuclease Activity Assay

Preparation of DNA substrate was performed following the protocol described previously (29, 30). Briefly, flap substrates for the 5′–3′ nuclease assay were prepared by annealing a downstream oligonucleotide, template, and upstream oligonucleotide at molar ratio of 1:2:4. For annealing, oligonucleotides were placed in a solution containing 1 mmol/L ethylenediaminetetraacetic acid (EDTA) and 10 mmol/L Tris-HCl, pH 8.0, heated to 100°C for 5 minutes, and slowly cooled to room temperature.

### Oligonucleotide Sequence (5′–3′)

Downstream: biotin-AGGTCTCGACTAACTCTAGTCGTTGTTCCACCCGTCCACCCGACGCCACCTCCTG; Template: GCAGGAGGTGGCGTCGGGTGGACGGGATTGAAATTTAGGCTGGCACGGTC; Upstream: CGACCGTGCCAGCCTAAATTTCAATA.

### Nuclease Activity Assay

Nuclease activity assay was performed according to the method described by Masuda-Sasa (30). Biotin-labeled flap DNA substrates (500 fmol) were added to the reaction mixture including 50 mmol/L Tris-HCl (pH 7.5), 10 mmol/L MgCl_2_, 2 mmol/L DTT, 100 mmol/L NaCl, 0.25 mg/mL BSA with or without recombinant flag-tagged DNA2 (10 nmol/L) in the presence of either DMSO or DNA2 inhibitor d16 (20 µmol/L). All reactions were incubated at 37°C for 10 minutes, followed by the addition of 2X stop solution (95% formamide, 20 mmol/L EDTA, 0.1% bromophenol blue and 0.1% xylene cyanol). The nucleolytic products were boiled for 1 minute and subjected to electrophoresis using 15% denaturing gel containing 7 mol/L urea. The gel was transferred to nitrocellulose membrane by electroblotting, UV cross-linked and subjected to streptavidin/avidin conjugates detection.

### *In Vivo* Xenograft Experiments

All animal care and experimental procedures were performed in accordance with protocols approved by the BCM Institutional Animal Care and Use Committee (IACUC). A total of 2 × 10^6^ MDAH-2774 cells were suspended in 150 µL PBS and injected subcutaneously into the dorsal flanks of NOD *scid* IL2 receptor γ chain knockout (NSG) mice. After the tumors had grown to the appropriate size for the experiments, the mice were randomized to receive the treatment with DNA2 inhibitor d16 alone or a combination of d16 and talazoparib. In the experiment investigating the effect of d16 alone, animals received intraperitoneal injection of d16 (30 or 15 mg/kg) twice weekly or vehicle DMSO control. In another study assessing the effect of the combination of d16 and talazoparib, d16 (20 mg/kg) was injected intraperitoneally twice weekly, and talazoparib (0.5 mg/kg) in 10% Dimethylacetamide (DMAc)/4% DMSO/PBS was injected intraperitoneally daily, 5 days per week. The nontreated control group received a mixture of 10% DMAc/PBS and DMSO. Tumor volume was measured every 3 days using caliper and calculated as follows: volume = longest tumor diameter × (shortest tumor diameter)^2^/2. The evaluator was blinded to the treatments. Animals were euthanized and tumors were harvested on the indicated dates. On the basis of the size variation of the MDAH-2774 xenografts, power analysis estimated 5 mice per group for 80% statistical power to detect 20% differences in tumor size between groups at *P* < 0.05. All experiments were performed under a BCM IACUC-approved protocol and all experiments confirm to IACUC standards and ethical regulations.

### Immunofluorescence Staining

MDAH-2774 cells were treated with DNA2 inhibitor d16 (10 µmol/L), RU (10 µmol/L), or both for 8 hours. Cells were fixed with 4% paraformaldehyde for 20 minutes, followed by permeabilization in PBS buffer containing 0.5% Triton X-100 and 0.5% NP-40 for 10 minutes. Cells were blocked in 2% BSA-containing PBS at room temperature for 1 hour, followed by incubation with anti-RAD51 rabbit mAb for 1 hour, and Texas Red X-conjugated anti-rabbit secondary antibody for another hour. Nuclei were stained with Hoechst 33258 dye. Images were captured with a Zeiss fluorescence microscope equipped with ApoTome 2 (Axio Observer inverted microscope).

### Traffic Light Reporter Assay

The reagents and constructs for traffic light reporter (TLR) system have been kindly provided by Dr. Scharenberg through Addgene. Accession codes: Addgene: 31475 (pCVL SFFV d14GFP donor), 31476 [pCVL SFFV d14GFP EF1s HA.NLS.Sce(opt)], [pCVL SFFV-EF1s HA.NLS.Sce(opt)]. The experimental procedure was performed as described by Certo and colleagues (31). Briefly, the TLR reporter construct contains an I-SceI nuclease cut site in an eGFP followed by an out-of-frame mCherry expressed in the event of a non-homologous end joining repair event. Upon HR repair with an exogenous donor template, the HR-repaired eGFP can produce green fluorescence, while keeping mCherry out of frame. HEK293T cells (RRID:CVCL_HA71) were cotransfected with the three constructs using standard PEI transfection protocol. Cells were then treated with d16 (20 µmol/L) or DMSO 48 hours following transfection. The cells were washed 24 hours after treatment, and positive cells for each construct were determined by flow cytometry and analyzed using FlowJo software (RRID:SCR_008520).

### Cellular Thermal Shift Assay

MDAH-2774 cells were harvested and resuspended in complete DMEM. Cell suspensions were treated with 20 µmol/L d16 in a CO_2_ incubator at 37°C for 1 hour. The cell suspensions were collected and washed with PBS three times and resuspended in PBS supplemented with protease inhibitor cocktail. The cells were then divided into smaller (100 µL) aliquots and heated individually at different temperatures ranging from 40°C to 45°C for 3 minutes followed by cooling for 3 minutes at room temperature. The cell suspensions were freeze-thawed three times using liquid nitrogen. The soluble fraction (lysate) was separated from the cell debris by centrifugation at 20,000 × *g* for 20 minutes at 4°C. The supernatants were transferred to new microtubes and analyzed by SDS-PAGE followed by Western blot analysis. For xenograft experiments, the frozen tumors were thawed on ice, and homogenized in cold PBS followed by three cycles of freeze-thawing using liquid nitrogen. Tissue lysates were separated from the cellular debris by centrifugation at 20,000 × *g* for 20 minutes at 4°C. The supernatants were diluted with PBS supplemented with protease inhibitor cocktail, aliquoted, and then heated at different temperatures for 3 minutes, followed by cooling down for 3 minutes at room temperature. Soluble fractions were isolated by centrifugation and analyzed as mentioned above.

### Statistical Analysis

Data were expressed as mean ± SD from at least three biological replicates. Statistical analyses were conducted using unpaired, two-tailed *t* test where *P* values < 0.05 were considered significant.

### Data Availability Statement

The data generated in this study are available within the article and its Supplementary Data. All other raw data are available upon request from the corresponding author.

## Results

### DNA2 Expression is Increased in Tumors with *TP53* Mutations Across Diverse Cancers

**FIGURE 1:**
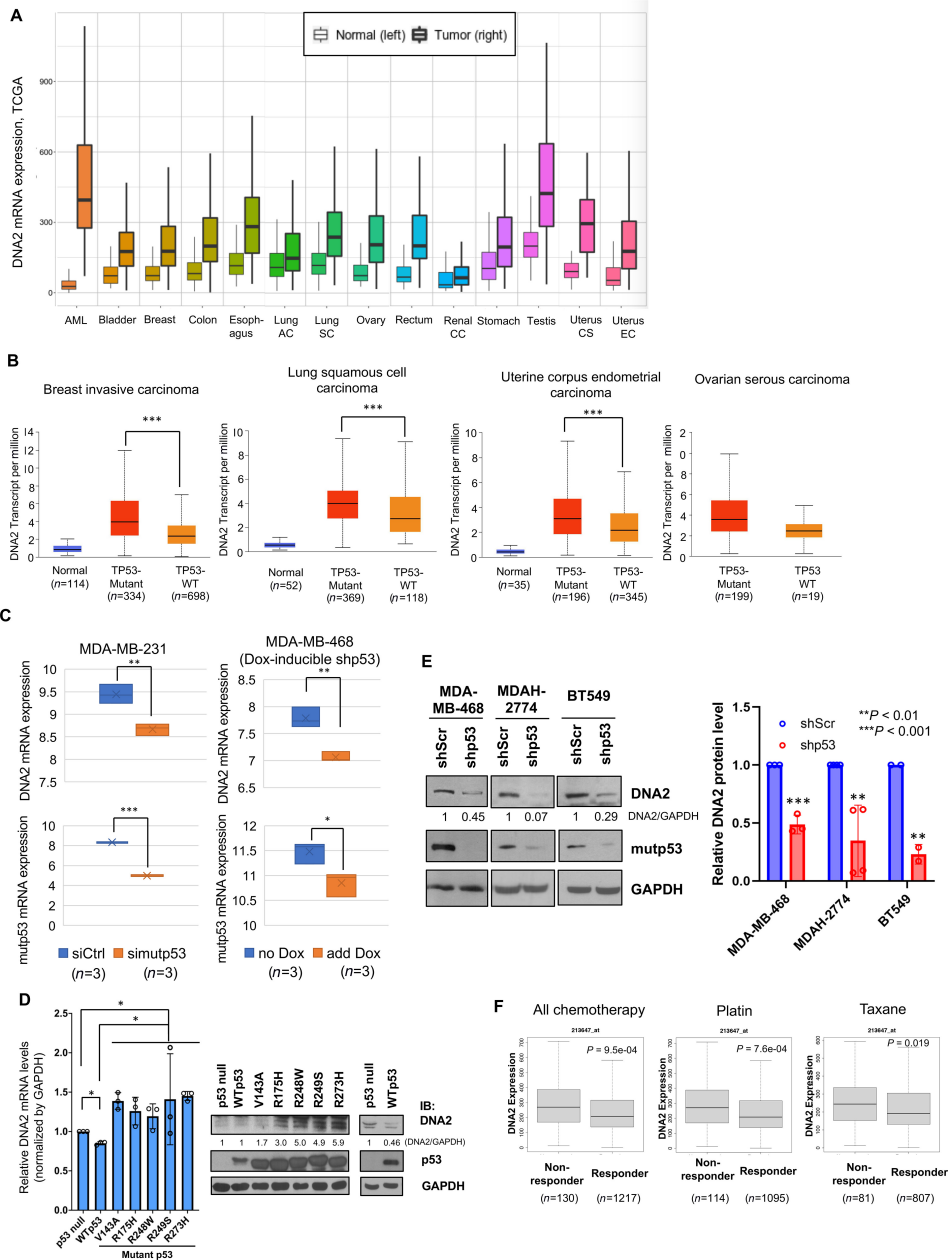
DNA2 is upregulated in many types of cancer, which is at least in part mediated by mutant p53. **A,** Expression of DNA2 is significantly elevated in 14 cancers from TCGA database compared with normal tissues. Tumor samples are denoted with bolded lines. Significance is calculated by Mann–Whitney *U* test. AC, adenocarcinoma; SC, squamous cell carcinoma; CC, clear cell; CS, carcinosarcoma; EC, endometrial carcinoma. **B,** Expression of DNA2 in breast invasive carcinoma, lung squamous carcinoma, uterine corpus endometrial carcinoma, and ovarian serous carcinoma from TCGA dataset based on *TP53* mutation status. The data from other types of cancer are shown in Supplementary Fig. S1. WT: wild type. **C,** Depletion of mutp53 decreased DNA2 mRNA expression in MDA-MB-231 and MDA-MB-468 cells. Gene expression data were extracted from GSE26262 (32) and GSE31812 (33), in which gene expression was performed using Affymetrix microarray and normalized using the Robust Multichip Average (RMA) algorithm (34). In MDA-MB-468 cells, the short hairpin RNA (shRNA) against mutp53 was induced by doxycycline (Dox). **D,** DNA2 mRNA and protein expression in p53-null H1299 cells transfected with either an empty vector, WT p53, or indicated mutp53 (V143A, R175H, R248W, R249S, and R273H). Forty-eight hours after transfection, DNA2 mRNA was measured by qRT-PCR and normalized by GAPDH. *, *P* < 0.05; **, *P* < 0.01; and ***, *P* < 0.001 indicating statistically significant difference (two-tailed *t* test). Right: p53 and DNA2 immunoblots. The relative intensities of DNA2 protein were quantified by NIH ImageJ software, and normalized by loading control GAPDH. **E,** Depletion of mutant p53 decreases DNA2 protein levels. MDA-MB-468, MDAH-2774 or BT549 cells stably expressing either a scrambled shRNA or a mutp53 shRNA were harvested and the total cell lysates were subjected to immunoblotting using antibodies specific to DNA2, p53 or GAPDH. The relative intensities of DNA2 were quantified by NIH ImageJ software, and normalized by loading control GAPDH. The right panel shows the fold difference of DNA2 protein in MDA-MB-468 (*n* = 3 biological replicates), MDAH-2774 (*n* = 4 biological replicates), or BT549 (*n* = 2 biological replicates) stably expressing a scrambled shRNA versus a mutp53 shRNA. **F,** Box plots of DNA2 mRNA expression in chemotherapy responder or nonresponder patients (defined by relapse-free survival at 6 months) of serous ovarian cancer. Data were analyzed from TCGA dataset using ROC plotter platform. Left: all chemotherapy; middle: platin; right: taxane.

According to TCGA Pan-Cancer database, DNA2 mRNA expression is upregulated in many types of cancer including acute myeloid leukemia, bladder, breast, colon, esophageal, lung, ovarian, rectal, stomach, and uterine cancers, etc. (Fig. 1A). These data suggest that DNA2 is commonly upregulated in cancer and may be considered as a cancer therapeutic target. Analysis of DNA2 expression profile in normal tissues and tumor samples with or without *TP53* mutations across different cancer types further shows that DNA2 is expressed at a higher level in tumor samples harboring mutant *TP53* than those harboring WT *TP53* across all of TCGA cancer types (Fig. 1B; Supplementary Fig. S1). Changes of DNA2 expression are statistically significant except in ovarian cancer, which is likely due to the low number of patients with WT *TP53*. These data suggest that high levels of DNA2 are linked to *TP53* mutations in cancer. To further investigate the effect of mutp53 on DNA2 expression, we also analyzed two microarray datasets GSE26262 (32) and GSE31812 (33) in which mutp53 was depleted in MDA-MB-231 and MDA-MB-468 cells, respectively. Indeed, depletion of mutp53 in MDA-MB-231 (harboring mutp53-R280K) or MDA-MB-468 (harboring mutp53-R273H) breast cancer cells attenuates DNA2 mRNA expression (Fig. 1C), supporting a role of mutp53 in DNA2 upregulation.

To further investigate a role for mutp53 in the overexpression of DNA2 in cancer cells, we measured the levels of DNA2 mRNA and protein after transfection of either WT p53 or one of the p53 hotspot mutants, including V143A, R175H, R248W, R249S, and R273H, in H1299 p53-null non–small cell lung cancer cell line. Strikingly, qRT-PCR analysis and immunoblotting showed that the levels of DNA2 mRNA and protein were elevated by overexpression of each hotspot mutp53 (Fig. 1D). In contrast, expression of WT p53 slightly decreased the expression of DNA2 mRNA, and either did not alter or slightly decreased the level of DNA2 protein. To further validate the effect of mutp53 on DNA2 expression, we depleted mutp53 in various mutp53-bearing cancer cell lines, including MDA-MB-468, MDAH-2774 (ovarian cancer cell line harboring mutp53-R273H), and BT549 (breast cancer cell line harboring mutp53-R249S). Consistently, depletion of mutp53 led to the decreased DNA2 protein expression in all three cell lines (Fig. 1E). These data demonstrate a role for mutp53 in upregulating DNA2 expression in cancer.

### Higher DNA2 Expression is Associated with Worse Clinicopathologic Characteristics in EOC

To assess the clinical significance of DNA2 in patients with mutant *TP53*-harboring cancers, we focused our data mining analysis on ovarian cancer as it is the cancer type with the most prevalent somatic *TP53* mutations. Nearly all (96%) high-grade serous ovarian carcinomas bear *TP53* mutations (35). We explored the clinical significance of DNA2 expression in ovarian cancer using the transcriptomic microarray database, CSIOVDB (26). Assessment of DNA2 expression profile according to the disease state shows that DNA2 levels are upregulated in ovarian carcinoma samples and in fallopian tube tumor samples compared with its counterparts, normal ovarian surface epithelium (*P* = 5.40e-12) and normal fallopian tube (*P* = 0.00217), respectively (Supplementary Fig. S2A). There is no significant difference between ovarian cancer stroma and its normal counterpart. Neither peritoneal nor other metastatic samples display differential expression of DNA2 compared with primary ovarian carcinoma. From a histologic perspective, overall, the ovarian cancer subtypes with less favorable prognosis, such as clear cell, endometrioid, and high-grade serous adenocarcinoma, express higher DNA2 levels than the less aggressive carcinoma, such as mucinous and tumors of low malignancy potential (*P* < 0.001; Supplementary Fig. S2B). Accordingly, the more metastatic and aggressive high-grade and late-stage ovarian cancer express significantly higher DNA2 levels (Supplementary Fig. S2C and S2D). To further investigate whether DNA could be a potential therapeutic target in ovarian cancer, we analyzed DNA2 expression profile in EOC molecular subtypes reported by Tan and colleagues (36). It is well known that EOC exhibits diverse outcomes and low survival rate even after the same or very similar regimen within one histologic type. To address this high degree of heterogeneity, a classification system was developed on the basis of gene expression patterns of 1,538 tumors, differentiating five biologically unique subgroups, namely epithelial-A (Epi-A), epithelial-B (Epi-B), mesenchymal (Mes), stem-like-A (Stem-A), and Stem-B. Noticeably, Stem-A and Mes subtypes had poor survival compared with Epi-A, Epi-B, and Stem-B (36). As shown in Supplementary Fig. S2E, DNA2 expression is higher in the Stem-A subtype that shows less favorable prognosis (*P* < 0.001) compared with all other subtypes. Notably, ovarian cancers that do not respond to chemotherapy such as platins or taxanes, express higher levels of DNA2 than those that respond in TCGA ovarian cancer cohort (*P* = 9.5e-04; Fig. 1F). In addition, survival analysis in all patients reveals that high DNA2 expression is linked to poor prognosis in overall survival (*P* = 0.002) and disease-free survival (*P* = 0.0175; Supplementary Fig. S2F). Within Stem-B subtype, a subtype with an overall better prognosis, DNA2 expression had a discriminating power, and high levels of DNA2 expression are associated with poor outcome in overall survival (*P* = 0.0439) and disease-free survival (*P* = 0.008; Supplementary Fig. S2G). These data suggest that high expression of DNA2 is associated with more advanced diseases, poor prognosis, and acquired resistance to therapies. Stem-A subtype is associated with much shorter survival compared with Stem-B (Supplementary Fig. S2H). Most ovarian cancers in Stem-A subtype overexpress DNA2; therefore, high DNA2 expression only has a trend, although not statistically significant, toward shorter survival (Supplementary Fig. S2H). To account for differences in stage and grade, we further separated survival according to stage and grade (Supplementary Fig. S2I–S2L). High DNA2 expression is associated with shorter progression-free survival in ovarian tumors, regardless of tumor stage or grade. Consistent with the result in Supplementary Fig. S2H, it also appears that DNA2 expression has a better discriminating power in early-stage or lower grade tumors. Besides the overexpression of DNA2 mRNA in mutp53-expressing cancer, it is evident that DNA2 protein is also overexpressed at least in lung squamous cell carcinoma and ovarian cancer (Supplementary Fig. S3A–S3D and S3F). The expression of DNA2 protein is correlated with DNA2 mRNA in lung squamous cell carcinoma (Supplementary Fig. S3E). Some tumors do show discordance of DNA2 expression between mRNA and protein, raising the possibility of additional mechanism(s) of regulation, such as protein stability, etc.

### DNA2 Inhibitor C5-derived Analogs Display Antitumor Activity in Human Cancer Cells with Mutant p53

**FIGURE 2:**
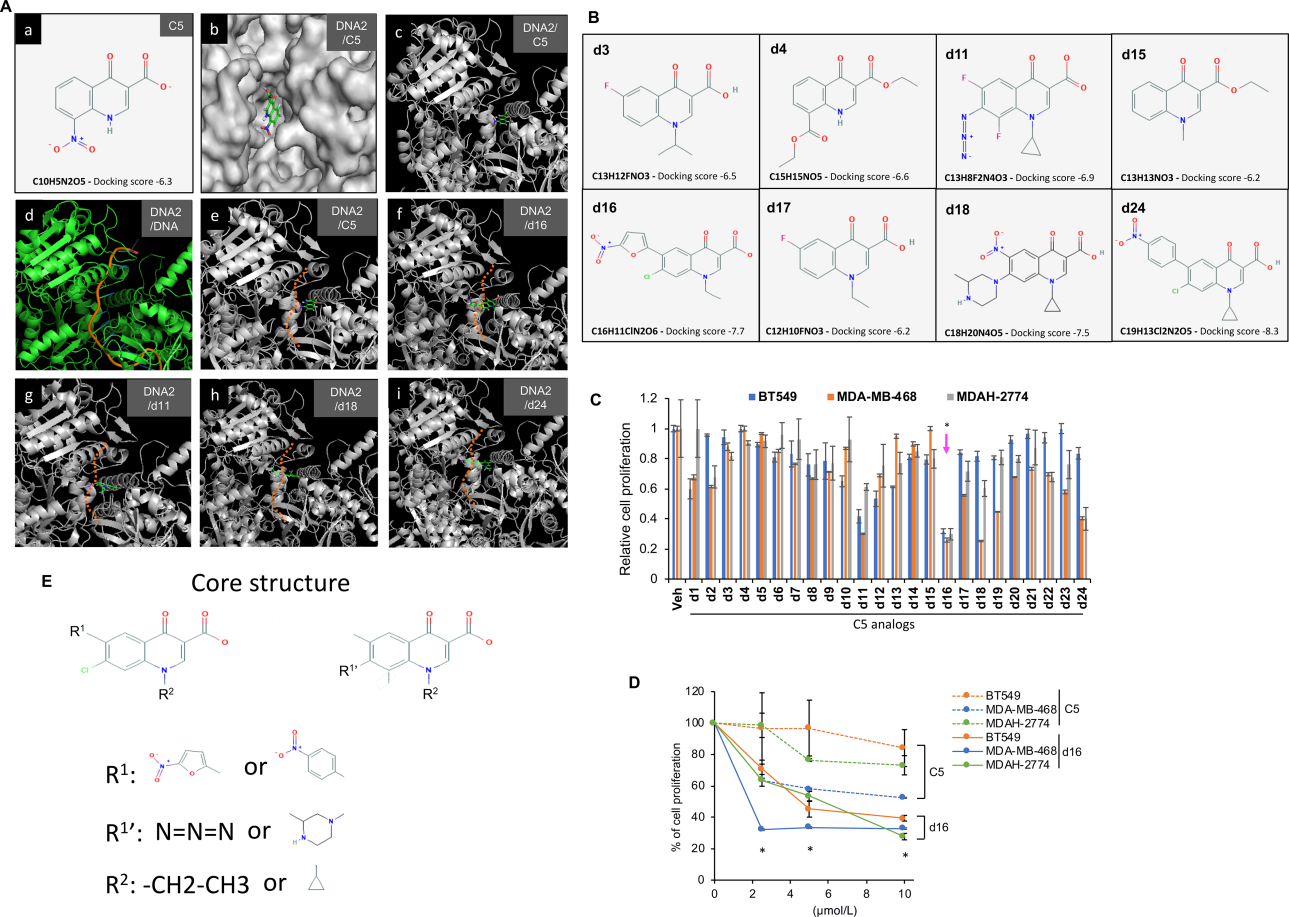
Cytotoxic effects of DNA2 inhibitor C5 and its analogs in human cancer cells. **A,** Molecular docking of C5 or its analog d16 in the Site 1 pocket of DNA2. A, C5 chemical structure and its docking score. **B** and **C,** The three-dimensional structure of DNA2 with C5 docked in the Site 1 pocket, surface view and cartoon view. **D,** Crystal structure of DNA2 in complex with an ssDNA (PDB: 5EAX; ref. 37). **E,** The positions of DNA and C5 in DNA2. **F–I,** The lowest-energy docking positions of d16, d11, d18, and d24 in DNA2 show potential interference with the passage of DNA strand (orange dotted line). B, Chemical structures of C5 analogs and their docking scores in DNA2. MDA-MB-468, BT549, and MDAH-2774 cells were treated for 72 hours with 20 µmol/L of one of the 24 C5 analogs (C) or with increasing concentrations of C5 or d16 (D). Cell proliferation was measured by MTT assay. The data shown represent mean ± SD (*n* = 3 biological replicates). *, *P* < 0.05 indicating statistically significant difference between vehicle (Veh) and C5 analogs (two-tailed *t* test). E, Structure-activity relationship analysis identifies the core structure of the active compounds.

Previously, a DNA2 inhibitor C5 (NSC-15765) was identified through a virtual high-throughput screening using a predicted structure of DNA2 helicase domain, which was built through homology modeling from the crystal structures of yeast and human Upf1 (21). In the Upf1-based DNA2 model structure, Liu and colleagues identified three druggable docking pockets (Sites 1, 2, and 3). They then used Site 1 that makes close contact with DNA for molecular docking and identified C5 (21). The IC_50_ of C5 for inhibiting DNA2 activity is 20 µmol/L. It is not very effective by itself in most cancer cells, leaving room for optimization of the compound. The crystal structures of mouse and human DNA2 were later published (37). We first assessed the docking of C5 into the published DNA2 structure. As shown in Fig. 2A(a–e), C5 can be docked to this pocket, and the binding of C5 is expected to interfere with DNA binding as confirmed previously (21). However, the docking score in the crystallized structure is only −6.3, as opposed to −8.3 in the modeled DNA2 structure (21). The low score might explain its low potency. We sought to identify C5 analogs with modifications of its side chains, such that it would adopt a more favorable hydrophobic interaction with the surrounding hydrophobic residues on both sides or create a better physical barrier to DNA binding by DNA2. We searched several compound databases, including ZINC and PubChem for C5 analogs with desired side-chain modifications and identified 24 C5 analog compounds (Supplementary Table S1). Figure 2B shows a few examples of the chemical structures and their corresponding docking scores in DNA2. We then compared the antitumor activity of these compounds across mutp53-expressing cell lines including the triple-negative breast cancer cells, MDA-MB-468 and BT549, and the ovarian cancer cells MDAH-2774. We assessed cell proliferation by MTT assay after 72 hours treatment. Among the 24 compounds, a C5 analog, namely d16, showed the best inhibitory effect on cell proliferation in all three cancer cell lines (Fig. 2C). Moreover, d16 showed a better potency than C5 across all three cancer cell lines (Fig. 2D). Other compounds, including d11, d18, and d24, also significantly decreased cancer cell viability, although their activities were lower than d16 (Fig. 2C). Comparison of C5 and d16 in DNA2 docking showed a more favorable docking position for d16 in the interference of DNA binding [Fig. 2A(e) vs. 2A(f)]. These findings suggest that d16 is a potential DNA2 inhibitor displaying potency in mutp53-harboring cancer cells. Docking of the other active compounds, that is, d11, d18 and d24, also shows interference of DNA2 binding to DNA [Fig. 2A(g–i)], suggesting that interference of DNA2 contact with DNA is a common feature of these active compounds. These active compounds also share a common core structure (Fig. 2E).

### Treatment with Compound d16 Induces Apoptosis and Cell-cycle Arrest

**FIGURE 3:**
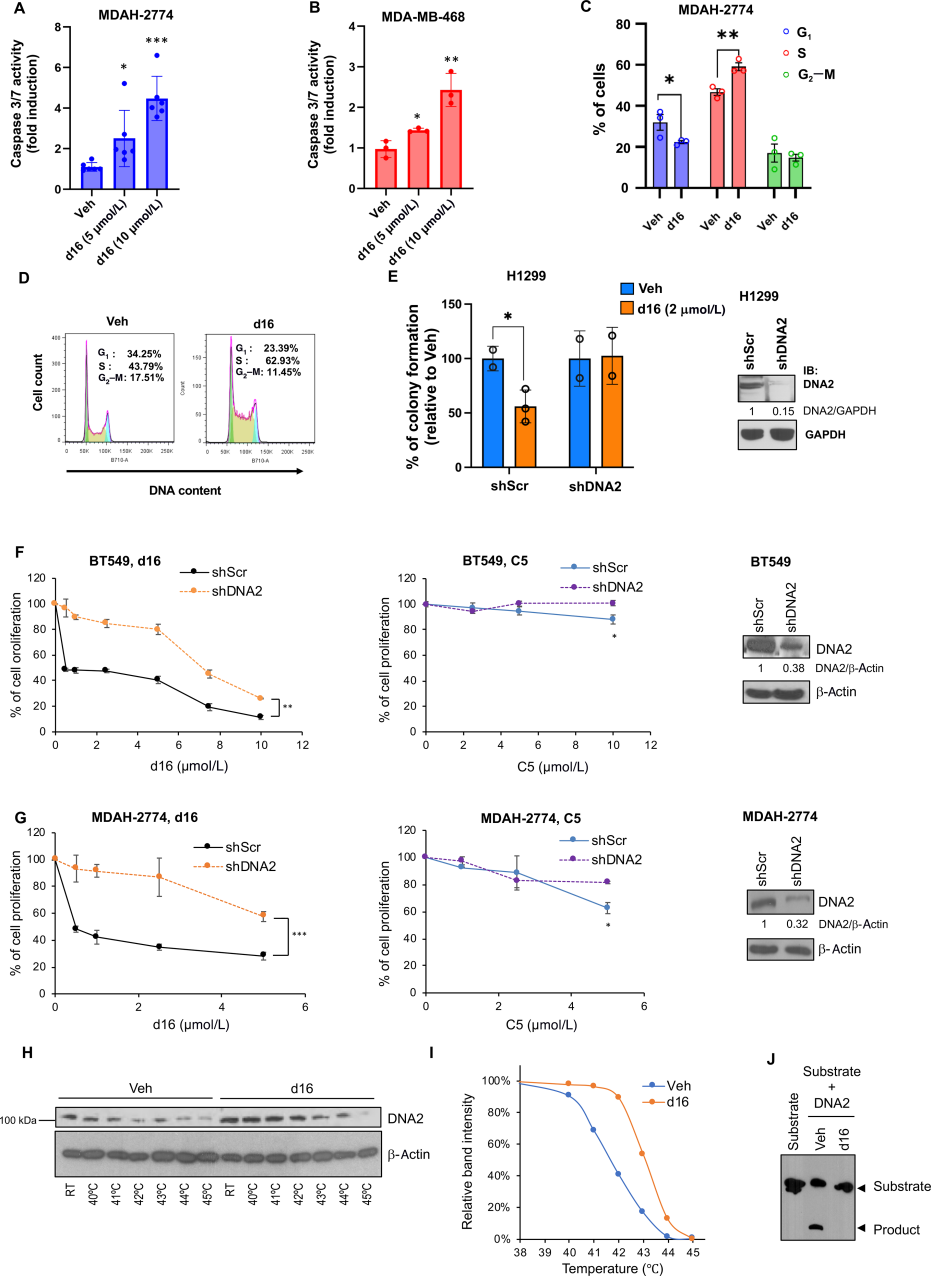
d16 displays on-target effects on DNA2 in mutp53-harboring breast and ovarian cancer cells. Cpd d16 modestly induces apoptosis in MDA-MB-468 (**A**) and MDAH-2774 cells (**B**). Cells were treated with DMSO vehicle or d16 (5 or 10 µmol/L) for 21 hours. Active caspase-3/7 was determined by Caspase-Glo 3/7 Assay, and was normalized to protein concentrations. The data shown represent the mean ± SD in MDA-MB-468 (*n* = 6 biological replicates) or MDAH-2774 (*n* = 3 biological replicates). *, *P* < 0.05; **, *P* < 0.01; and ***, *P* < 0.001 indicating statistically significant difference (two-tailed *t* test). **C** and **D,** Flow cytometric cell cycle analysis of MDAH-2774 cells treated with vehicle (Veh) or 20 µmol/L d16 overnight. **C,** Quantification of cells stained with PI indicates the percentage of cell distribution in G_1_-, S-, and G_2_–M-phase. **D,** Representative PI profiles. *, *P* < 0.05; **, *P* < 0.01 (two-tailed *t* test, *n* = 3 biological replicates). **E,** Colony formation of H1299 stable cells harboring a DNA2 shRNA (shDNA2) or a scrambled shRNA (shScr). Cells were treated with vehicle (Veh) or 2 µmol/L d16 for 24 hours and cultured for additional 7 days in a drug-free medium. Bar chart shows the percentage of colonies relative to untreated cells of each group. Error bars = mean ± SD; *n* = 3; *, *P* < 0.05. Right: Western blotting confirms DNA2 knockdown in H1299 cells. BT549 (**F**) and MDAH-2774 (**G**) stable cell lines expressing a scrambled shRNA (shScr) or a DNA2 shRNA (shDNA2) were treated with increasing concentrations of d16 or C5 for 72 hours followed by MTT assay. Cell lysates of both cell lines were analyzed by immunoblotting (right). The data shown represent mean ± SD (*n* = 3 biological replicates). *P* values are *, *P* < 0.05; **, *P* < 0.01; and ***, *P* < 0.001 (two-tailed *t* test). CETSA, Western blot (**H**) and melt curves (**I**) from MDAH-2774 cell lysates following d16 (20 µmol/L) treatment. The intensity of DNA2 was quantified using NIH ImageJ software, normalized by its corresponding β-actin signal, and relative to the room temperature) sample. **J,** Recombinant flag-tagged DNA2 (10 nmol/L) was mixed with biotin-conjugated flap DNA substrate (500 fmol) in the absence or presence of d16 (20 µmol/L). The image shows a representative result of biochemical reaction (37°C, 30 minutes) that was resolved using 15% denaturing polyacrylamide gel electrophoresis.

To determine the mechanisms by which d16 inhibited cancer cell proliferation, we first determined the effect of d16 on apoptosis by measuring the caspase-3/7 activity in MDAH-2774 and MDA-MB-468 cells. As shown in Fig. 3A and B, d16 induced caspase-3/7 activation in a dose-dependent manner, indicating an induction of apoptosis. We next examined the effect of d16 on the cell-cycle profile in MDAH-2774 cells. It appears that d16 increased the proportion of cells in S-phase, but decreased that in G_1_-phase (Fig. 3C and D), indicating an arrest in the S-phase. The changes of cell-cycle profile in MDAH-2774 cells are very similar to that found in U2OS cells where depletion of DNA2 causes a reduction of cell population at G_1_-phase and an arrest at late S–G_2_-phase, as reflected by a reduction of G_1_/(S+G_2_) ratio (38). Consistent with the depletion of DNA2 (38), d16 also caused a reduction of G_1_/(S+G_2_) (Supplementary Fig. S4A). Interestingly, a short pulse of BrdU labeling showed that d16 treatment increased BrdU(+) cell population in MDAH-2774 cells (Supplementary Fig. S4B). Because inhibition of DNA2 can synergize with mutp53 to inhibit ATR function (9), it is likely that this result reflects an inhibition of ATR checkpoint in MDAH-2774 cells that harbor mutp53.

### Compound d16 Targets DNA2 to Elicit the Antitumor Activity

To determine whether d16 targets DNA2 within the cells, we depleted DNA2 in H1299 cells and performed clonogenic survival assay after d16 treatment. Depletion of DNA2 blocked the effect of d16 on the inhibition of clongenic viability in H1299 cells (Fig. 3E). This result strongly supports DNA2 as the cellular target of d16. To determine whether the effect of d16 was specific to DNA2 in mutp53-bearing cancer cells, we first measured the antitumor activities of d16 and used C5 as a reference in control and DNA2-knockdown BT549 and MDAH-2774 stable cell lines. We found that knockdown of DNA2 attenuated the inhibitory effect of d16 or C5 on cell proliferation in both BT549 and MDAH-2774 cell lines (Fig. 3F and G). In addition, d16 demonstrated a much better potency than C5. To further validate the targeting of DNA2 by d16, we assessed the ability of d16 to bind endogenous DNA2 in live cells by performing a cellular thermal shift assay (CETSA; refs. 39, 40). CETSA showed that d16 treatment in MDAH-2774 cells shifted the melting temperature (Tm) of DNA2 protein toward a higher temperature, providing evidence for the binding of d16 to DNA2 inside the cells (Fig. 3H and I). In contrast, d4, an inactive C5 analog, failed to shift the Tm of DNA2 protein (Supplementary Fig. S5). Furthermore, to investigate the effect of d16 on DNA2 endonuclease activity, we used recombinant human DNA2 to cleave a well-defined flap substrate as described in Kim and colleagues (29) and found that in the presence of d16, DNA2 failed to cleave the substrate (Fig. 3J). These results suggest that d16 effects are specific and mediated by targeting DNA2.

### DNA2 Inhibition and mutp53 Expression are Synthetic Lethal by Impairing ATR Function in Cancer Cells

**FIGURE 4:**
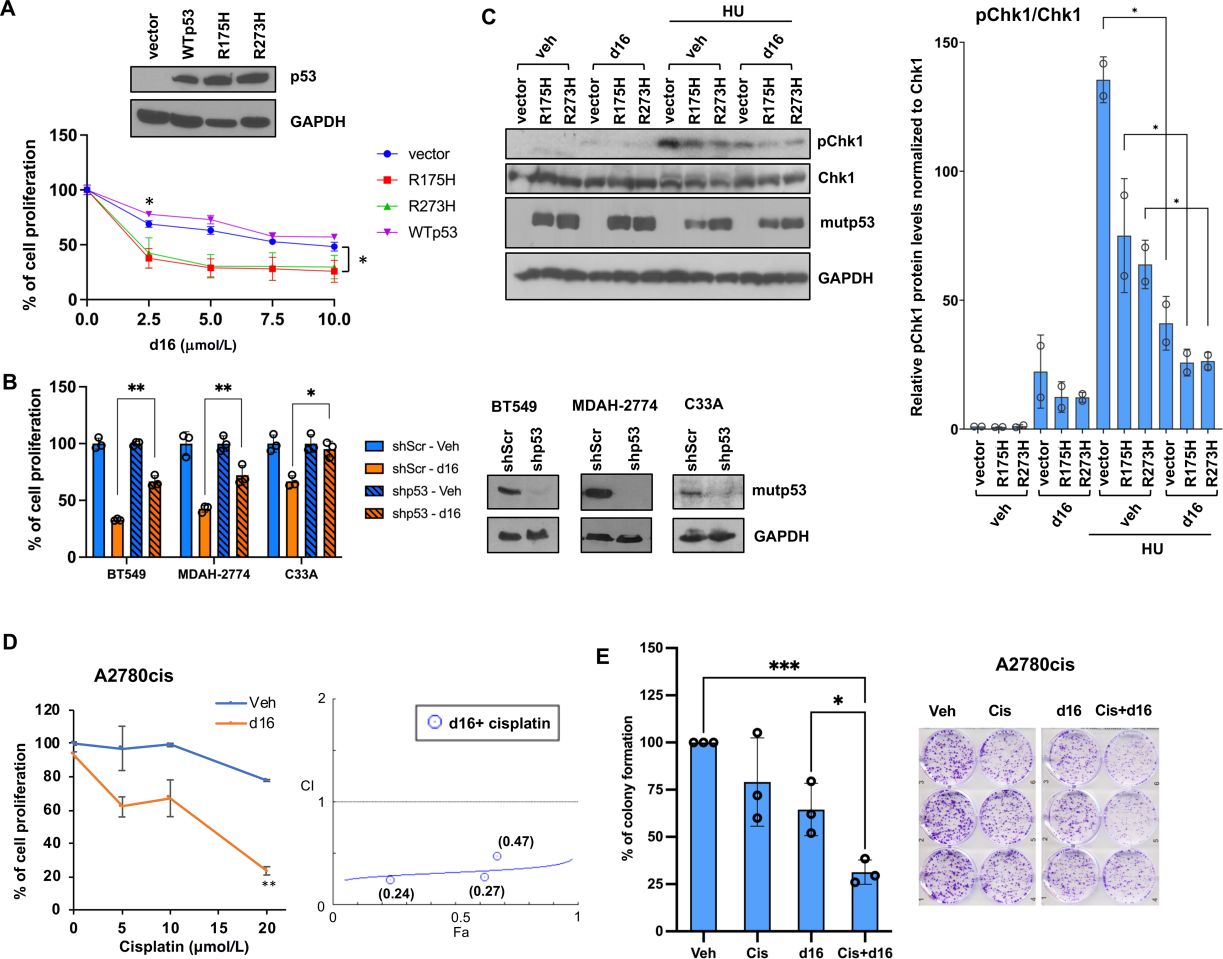
Targeting DNA2 with d16 inhibits ATR function and enhances cisplatin sensitivity in cancer cells harboring mutp53. **A,** H1299 stable cells expressing an empty vector, WT p53, mutp53-R273H or mutp53-R175H were treated for 72 hours with increasing concentrations of d16. Cell proliferation was measured by MTT assay. Top panels are immunoblots showing the expression of WT p53, mutp53, and GAPDH, respectively. **B,** MDAH-2774, BT549, and C33A cells stably expressing either a scrambled shRNA (shScr) or an shRNA against mutp53 (shp53) were treated with d16 (5 µmol/L for BT549 and MDAH-2774, 2.5 µmol/L for C33A) for 72 hours. Cell viability was then measured by MTT assay. The data shown represent mean ± SD (*n* = 3 biological replicates). *P* values are *, *P* < 0.05; **, *P* < 0.01; and ***, *P* < 0.001 indicating statistically significant difference (two-tailed *t* test). Right: Immunoblots of mutp53 and GAPDH, respectively. **C,** H1299 cells transiently expressing an empty vector or mutp53-R273H were treated with d16 (10 µmol/L) for 24 hours, followed by HU (2 mmol/L) treatment for 0, 2, or 4 hours. Cell lysates were subjected to Western blotting. The right panel shows the quantification of pChk1 protein levels normalized by Chk1 protein; *, *P* < 0.05. **D,** A2780cis cells were treated with vehicle control or d16 (5 µmol/L) with increasing doses of cisplatin for 72 hours. Cell proliferation was then measured by MTT assay. CI analysis was performed using CompuSyn software. Fa (Fraction affected)-CI plot and CI values (in brackets) were calculated and shown in the right. CI < 1 indicates synergism. **E,** Clonogenic survival assay was performed by treating A2780cis cells with d16 (2.5 µmol/L), cisplatin (2 µmol/L), or both for 24 hours. After PBS washing, cells were cultured for 10 days. The data shown represent mean ± SD (*n* = 3 biological replicates). *, *P* < 0.05; **, *P* < 0.01; and ***, *P* < 0.001 indicating statistically significant difference (two-tailed *t* test).

Human DNA2 can activate the ATR checkpoint under replication stress in S-phase (10, 12). We previously reported that mutp53 interferes with ATR/TopBP1 binding to inhibit the ATR-activating function of TopBP1 and decrease the checkpoint response to replicative stress (9), leaving ATR function only partially effective. As a complete loss of ATR function leads to lethality (16), mutp53-bearing cancer cells are more vulnerable to DNA2 depletion or a DNA2 inhibitor C5 (9). These findings suggest a novel synthetic lethality strategy against mutp53-bearing tumors by targeting DNA2. To determine whether d16 and mutp53 have synthetic lethal interaction in cancer cells, we expressed mutp53-R175H, mutp53-R273H or WT p53 in p53-null cancer cell line H1299 and measured cell proliferation following d16 treatment. Indeed, expression of mutp53-R175H or mutp53-R273H, but not WT p53, enhanced the sensitivity to d16 in H1299 cells (Fig. 4A). Conversely, depletion of mutp53 in all three cancer cell lines, BT549, MDAH-2774, and C33A (harboring mutp53-R273C) reduced the sensitivity to d16 (Fig. 4B). We also added d16 to H1299 cells expressing mutp53-R273H or mutp53-R175H, and then assessed hydroxyurea (HU)-induced Chk1 activation to determine whether ATR/Chk1 function was further impaired. We found that HU-induced Chk1 activation was attenuated by either expression of mutp53 (R175H or R273H) or treatment with d16, and was further inhibited by the combination of both (Fig. 4C), demonstrating the synthetic lethal interaction between d16 treatment and mutp53 expression. Previously, we showed that depletion of DNA2 renders mutp53-expressing cancer cells hypersensitive to cisplatin (9). To investigate the effect of DNA2 inhibition on the rescue of chemotherapy resistance, we treated cisplatin-resistant ovarian cancer cells A2780cis (harboring mutp53-K351N; ref. 41) with d16 and/or cisplatin, and measured cell proliferation and clonogenic survival. MTT assay showed that d16 treatment resensitized A2780cis cells to cisplatin (Fig. 4D). To determine and quantify the dose effect of the combination between d16 and cisplatin, we used the CompuSyn software, a computational program based on the median-effect equation of Chou-Talalay for drug CI method (23). CI analysis revealed the quantification of drug interactions, where CI < 1, = 1, and >1 indicates synergism, additive effect, and antagonism, respectively (24). The quantitative analysis obtained by CompuSyn demonstrated that d16 and cisplatin have a synergistic effect (CI < 1; Fig. 4D, right). Likewise, a combination of cisplatin and d16 treatment further reduced clonogenic survival compared with d16 or cisplatin treatment alone (Fig. 4E). As high DNA2 expression is also associated with resistance to chemotherapy in ovarian cancer (Fig. 1F), these data suggest that DNA2 inhibitors may be employed as a therapeutic strategy to overcome chemotherapy resistance in mutant p53-harboring cancers.

### Treatment with DNA2 Inhibitor d16 Reduces Ovarian Cancer Growth *In Vivo*

**FIGURE 5:**
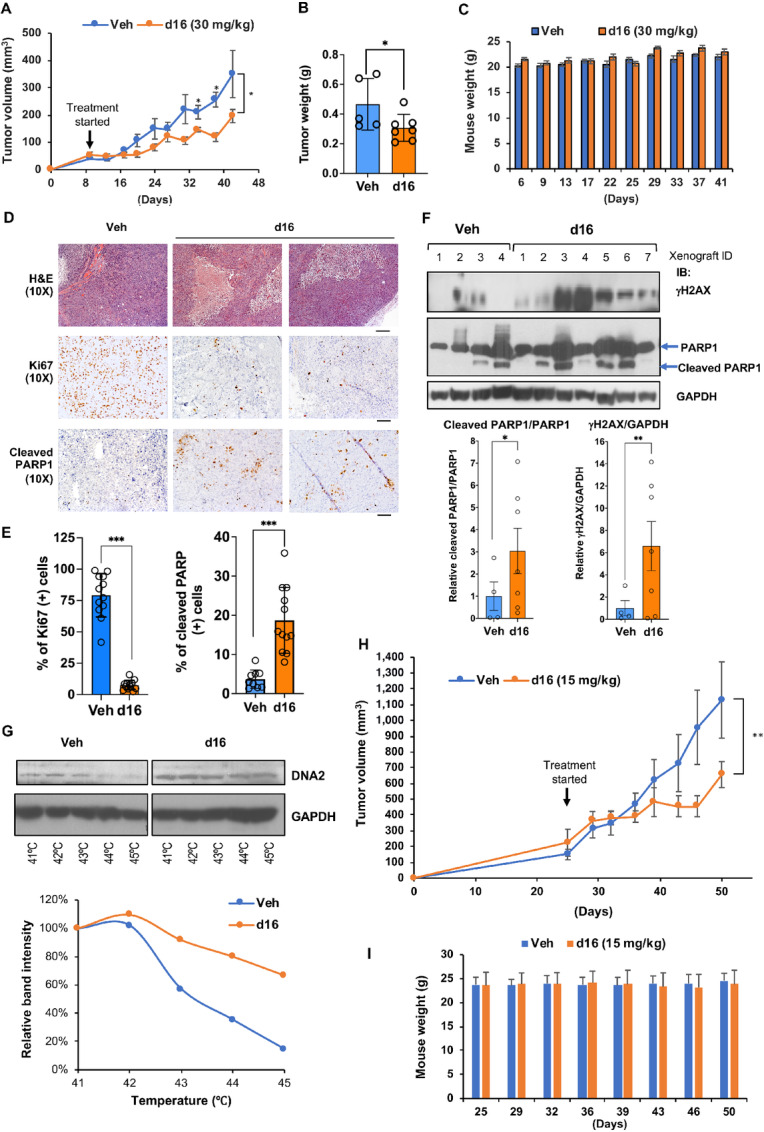
d16 treatment inhibits ovarian cancer growth *in vivo*. **A,** NSG mice bearing MDAH-2774 xenografts were administered with d16 (30 mg/kg, twice/week) or vehicle (Veh) via intraperitoneal injection for 1 month. Data shown are the mean tumor volumes ± SD; *n* = 5 (Veh), or 7 (d16). *, *P* < 0.05 (two-tailed *t* test). **B,** Tumor weight. **C,** The mean mouse body weight ± SD. There was no statistical difference between these two groups. **D,** Representative images of H&E, Ki-67, and cleaved PARP1 staining of xenograft tumors. Scale bar, 100 µm. **E,** Quantification is shown in bar graphs. ***, *P* < 0.001, comparing Veh and d16 groups (two-tailed *t* test). **F,** Western blot analysis of the xenograft lysates showed the induction of γH2AX and PARP1 cleavage in many of the d16-treated xenografts compared with control. The bottom panels show the quantification of cleaved PARP1/full-length PARP1 and γH2AX/GAPDH from four Veh-treated and seven d16-treated xenografts. *, *P* < 0.05; **, *P* < 0.01 (two-tailed *t* test). **G,** CETSA was performed using the lysates of MDAH-2774 xenografts from mice treated with vehicle DMSO or d16. **H,** NSG mice bearing MDAH-2774 xenografts were administered with d16 (15 mg/kg, twice/week) or vehicle (Veh) via intraperitoneal injection for 3–4 weeks. Data shown are the mean tumor volume ± SD; *n* = 7 per group. **, *P* < 0.01 (two-tailed *t* test). **I,** The mean mouse body weight ± SD. There was no statistical difference between these two groups.

To determine the antitumor activity of d16 *in vivo*, we established an ovarian cancer MDAH-2774 xenograft model in NOD scid IL2 receptor γ chain knockout mice (NSG) mice. The MDAH-2774 xenograft-bearing NSG mice were treated with vehicle control or d16 (30 mg/kg) twice a week by intraperitoneal injection for 1 month. As shown in Fig. 5A and B, d16 treatment effectively reduced tumor growth as shown by the decrease of tumor volume and final tumor weight. Mice tolerated d16 treatment very well, and there was no difference in the overall body weight between vehicle control and d16 treatment groups (Fig. 5C). To assess the antiproliferative effect of d16, we performed Ki67 IHC staining of tumor sections from both groups (Fig. 5D). The quantification showed that d16 treatment decreased the number of Ki67-positive cells compared with the control group (Fig. 5E). These results suggest that targeting DNA2 with d16 inhibits cell proliferation *in vivo*. We also assessed the proapoptotic effect of d16 by quantifying cleaved PARP1 IHC staining. We found that d16 treatment increased the percentage of cleaved PARP1-positive cells compared with the vehicle control group (Fig. 5D and E). Western blot analysis of tumor lysates further confirmed that PARP1 cleavage was increased by d16 treatment (Fig. 5F). Because DNA2 can prevent replication-associated DSBs (20), inhibition of DNA2 may cause DNA damage. Therefore, we next measured the level of phosphorylated H2AX (γH2AX). As shown in Fig. 5F, γH2AX was expressed at much higher levels in most of the d16-treated samples compared with the vehicle-treated samples. We also performed CETSA using the xenograft tumor tissues treated with vehicle or d16. Consistent with the results shown in Fig. 3H and I, d16 shifted the Tm of DNA2 protein, providing evidence for the binding of d16 to DNA2 *in vivo* (Fig. 5G). In Fig. 5A, the d16 treatment was initiated while the xenografts were relatively small (about 50 mm^3^). Here we repeated the experiment and started d16 treatment when the xenografts had grown to bigger sizes (150–250 mm^3^) and reduced the dosage of d16 to 15 mg/kg, twice weekly. Consistently, we observed similar reduction of tumor volumes by d16 treatment (Fig. 5H) without significant effect on mouse body weight (Fig. 5I).

Together, these data demonstrate the *in vivo* antitumor activity of d16 against a mutp53-bearing ovarian cancer xenograft model.

### DNA2 Inhibition Impairs the HR Repair Pathway and Synergizes with PARPis

**FIGURE 6:**
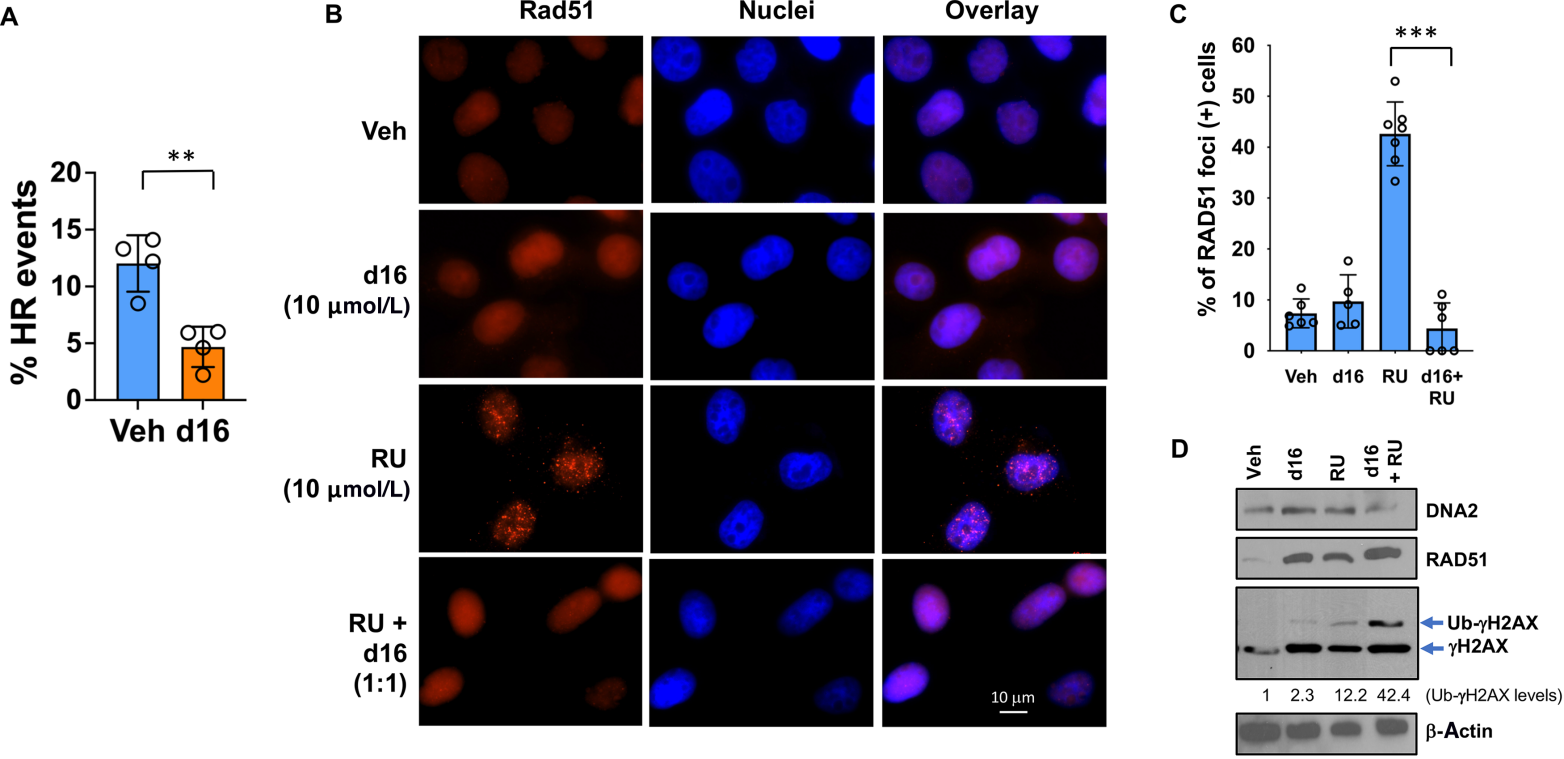
DNA2 inhibitor d16 decreases HR repair. **A,** Quantification of HR activity in HEK293T cells using the TLR assay after treatment with d16 (20 µmol/L). **, *P* < 0.01 (two-tailed *t* test). A representative set of flow profiles are shown in Supplementary Fig. S6. **B,** MDAH-2774 cells were treated with d16, RU or both for 8 hours. After fixation and permeabilization, immunostaining was performed with an anti-RAD51 rabbit antibody, followed by Texas Red X-conjugated anti-rabbit secondary antibody. Nuclei were stained with Hoechst 33258. Pictures shown are representative images at 100X magnification. **C,** Quantification of RAD51 foci-positive cells. ***, *P* < 0.001 (two-tailed *t* test). **D,** Immunoblotting shows the expression of DNA2, Rad51, γH2AX, or β-actin in the whole-cell lysates. The Ub-γH2AX signals were quantified using NIH ImageJ software.

**FIGURE 7:**
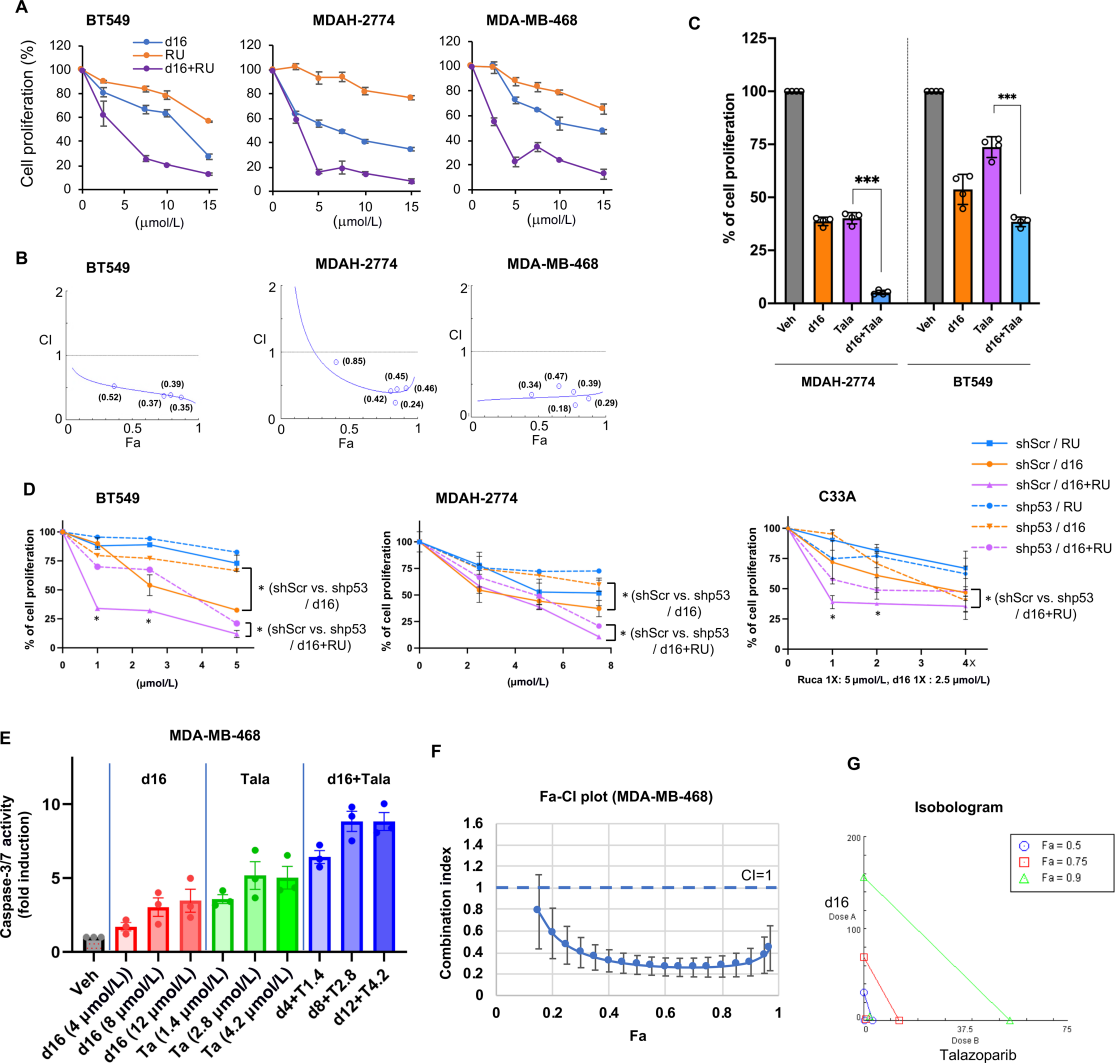
DNA2 inhibitor d16 synergizes with PARPis in cancer cells. **A,** BT549, MDAH-2774, and MDA-MB-468 cells were treated with increasing concentrations of d16, RU, or both as indicated. MTT assay was performed 72 hours following treatment. **B,** CI values were calculated (in brackets) and Fa-CI plots were generated using CompuSyn software. CI < 1 indicates synergism. The isobolograms for each CI analysis are shown in Supplementary Fig. S7A. **C,** MDAH-2774 and BT549 were treated with d16 (10 µmol/L), talazoparib (Tala; 2.5 µmol/L) or both for 72 hours, and cell viability was measured by MTT assay. **D,** MDAH-2774, BT549, and C33A cells stably expressing either a scrambled shRNA (shScr) or an shRNA against mutp53 (shp53) were treated with increasing concentrations of d16, RU, or both as indicated for 72 hours followed by MTT assay. The data shown represent mean ± SD (*n* = 3 biological replicates). *P* values are *, *P* < 0.05 and ***, *P* < 0.001 indicating statistically significant difference (two-tailed *t* test). Expression of mutp53 in these stable cell lines is shown in Fig. 4B. The detailed Fa-CI plots are shown in Supplementary Fig. S7B. **E–G,** Cpd 16 and PARP1/2 inhibitor talazoparib synergistically induce apoptosis in breast cancer cells. MDA-MB-468 cells were treated with increasing concentrations of d16, talazoparib, or both as indicated for 21 hours. Active caspase-3/7 was determined by Caspase-Glo 3/7 Assay and was normalized to protein concentrations. Data shown are the mean ± SE from three independent experiments. CI values, Fa-CI plots and Isobologram were generated using CompuSyn software. CI < 1 indicates synergism.

The helicase/nuclease function of DNA2 plays a role in HR repair by accumulating at the replication fork, resecting DNA DSBs, and recruiting RAD51 to repair DSBs (20, 42). We first determined whether d16 treatment affected the HR repair pathway using the traffic light reporter (TLR) assay described by Certo and colleagues (31). The TLR flow cytometric analysis showed that d16 treatment decreased the HR efficiency (Fig. 6A; Supplementary Fig. S6). We next quantified RAD51-induced foci formation following treatment with a PARPi RU, d16 or both. As shown in Fig. 6B and C, RU treatment increased RAD51 foci formation, while d16 treatment did not significantly affect RAD51 accumulation. Strikingly, RU-induced RAD51 foci formation was eliminated when combined with d16, suggesting that inhibition of DNA2 by d16 prevents HR repair and therefore blocks PARPi-induced RAD51 foci formation. It appears that the abrogation of RAD51 foci was not due to reduced RAD51 protein levels (Fig. 6D), but was resulted from the inhibition of RAD51 recruitment at the DNA DSB site to initiate repair. Treatment with d16 or RU also increased the level of γH2AX (Fig. 6D). Moreover, the combination of d16 and RU further increased the level of monoubiquitinated (Ub)-γH2AX, suggestive of a higher extent of DNA damage. Because PARP inhibition and HR deficiency are synthetically lethal (43), we expected that d16 treatment inhibits HR and therefore might enhance the sensitivity to PARPis. To assess the effect of combination of PARPi and d16 on cell survival, we measured the proliferation of breast cancer and ovarian cancer cells following treatment with d16, RU, or both. MTT assay showed that combination of d16 and RU further decreased cell proliferation compared with single treatment in breast and ovarian cancer cells (Fig. 7A). A CI analysis showed that d16 and RU exerted a synergistic effect in all three cell lines BT549, MDAH-2774, and MDA-MB-468 (Fig. 7B; Supplementary Fig. S7A). The synergy could also be observed between d16 and another PARPi talazoparib (Fig. 7C). We next determined the effect of mutp53 on the response to d16 and PARPi. Consistent with the result in Fig. 4B, depletion of mutp53 rendered cells more resistant to d16 in MDAH-2774, BT549 cells, and C33A cells (Fig. 7D, orange dotted line vs. solid line). Moreover, the response to the combination of d16 and RU was reduced upon depletion of mutp53 (Fig. 7D, pink dotted line vs. solid line), which is likely due to a less response to d16 in mutp53-depleted cells. The CI analysis shows that the synergy between d16 and RU was not disrupted by depletion of mutp53; however, the overall Fa-CI plots were shifted right, suggesting that higher doses of drugs are required to reach synergy (Supplementary Fig. S7B). To further examine the synergy of d16 with PARPi, we also performed caspase-3/7 activity assay in MDA-MB-468 cells treated with d16 and/or talazoparib. Indeed, d16 and talazoparib synergistically induced apoptosis in MDA-MB-468 cells (Fig. 7E–G).

### DNA2 Inhibitor d16 Synergizes with Talazoparib to Exert Antitumor Activity in Ovarian Cancer Xenografts

**FIGURE 8:**
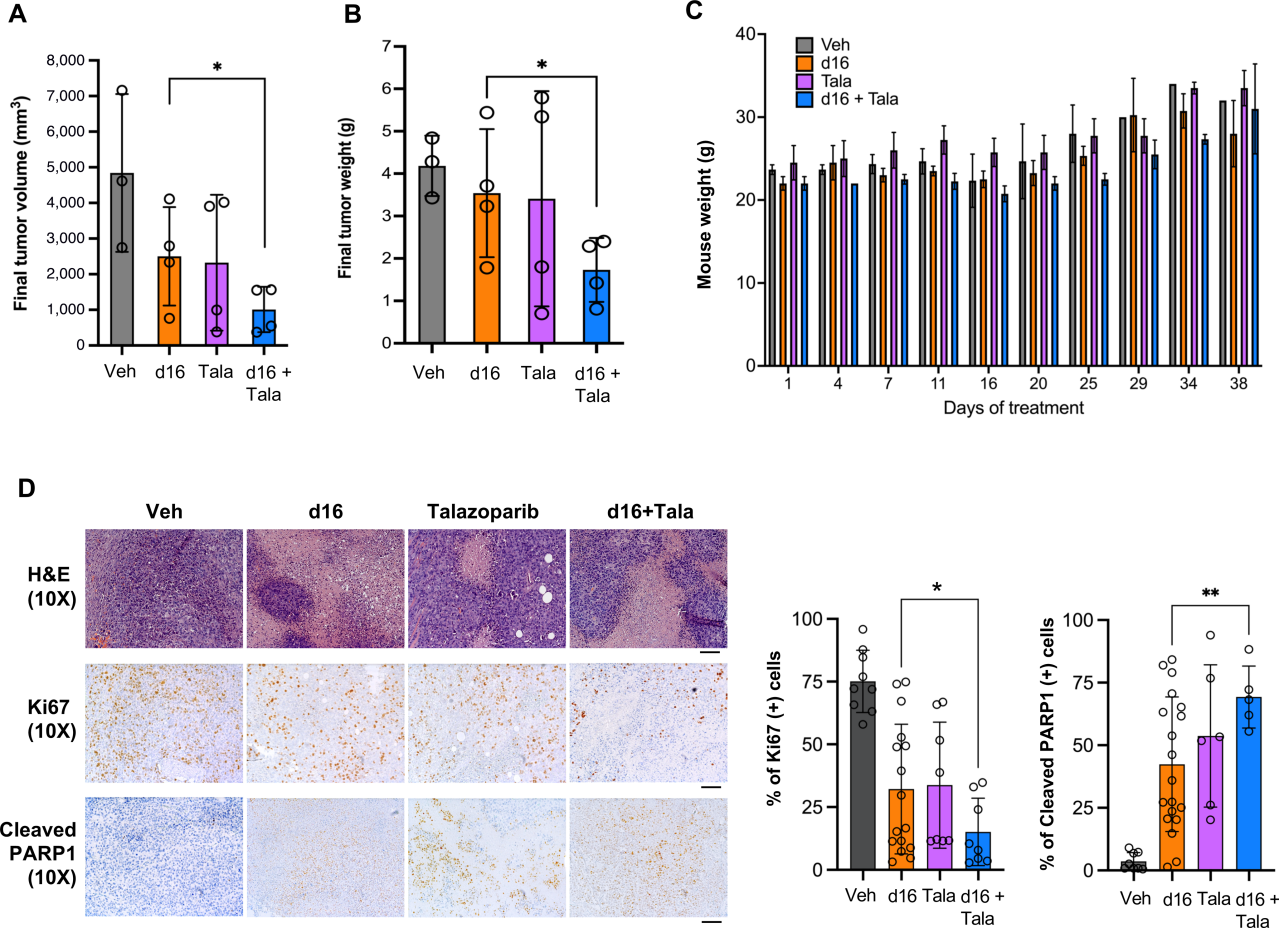
Combination of d16 and talazoparib treatment inhibits ovarian cancer growth *in vivo*. MDAH-2774 cells were subcutaneously implanted into the right flank of NSG mice. Xenografts were randomized into four groups: control (Veh), d16 (20 mg/kg, i.p., twice/week), talazoparib (Tala; 0.5 mg/kg, i.p., once daily, 5 days per week), or combination of d16 and talazoparib (*n* = 3 in Veh group, *n* = 4 in the other groups) for 5 weeks. **A,** Final tumor volume. **B,** Final tumor weight. **C,** Mouse body weight. **D,** Representative IHC images of Ki67 and cleaved PARP1 of xenograft tumor tissues. Quantification is shown in bar graphs. Scale bar, 100 µm. Results are presented as mean ± SD. *, *P* < 0.05; **, *P* < 0.01 (two-tailed *t* test).

To validate the synergistic effect of DNA2 inhibitor d16 and PARPi *in vivo*, we established ovarian cancer MDAH-2774 xenografts in NSG mice and treated the mice with vehicle control, d16 (20 mg/kg, i.p. injection, twice/week), talazoparib (0.5 mg/kg, i.p. injection, daily 5×/week), or both. Indeed, combination of d16 and talazoparib showed the best inhibitory effect on tumor growth (Fig. 8A and B). Mice tolerated the combination treatment without apparent toxicity or significant body weight loss (Fig. 8C). The IHC showed decreased proliferation and increased apoptosis in d16 or talazoparib-treated tumors, as indicated by the reduced Ki67 staining and elevated PARP cleavage (Fig. 8D). This effect was further enhanced by a combined treatment with d16 and talazoparib. These findings suggest that the DNA2 inhibitor d16 can synergize with PARPi talazoparib to serve as a potentially effective therapy for ovarian cancer.

## Discussion

### Structure-based Optimization and Development of DNA2 Inhibitors

Previously, using homology modeling of DNA2, Liu and colleagues recognized three well-defined pockets (Sites 1, 2, and 3) in DNA2, and subsequently identified C5 as a DNA2 inhibitor through the Site 1 molecular docking (21). Here we performed structure-based virtual screening and lead optimization by docking C5 into the Site 1 of the DNA2 crystal structure, aiming to identify C5 analogs with more favorable interactions with surrounding amino acids or capable of forming a better physical barrier to DNA binding. We identified several active compounds with a core structure distinct from C5 in the side chain positions, which lead to a better interference with DNA2 binding to DNA (Fig. 2). Among these compounds, d16 was identified and proved to be a more potent DNA2 inhibitor than C5 in blocking cell proliferation of various cancer types including ovarian, breast, cervical, and lung cancers. d16 also reduces tumor growth of ovarian xenografts and increases sensitivity to cisplatin in cisplatin-resistant ovarian cancer cells. Several lines of evidence support the targeting of DNA2 by d16: (i) d16 directly inhibits the nuclease activity of purified DNA2 protein in an *in vitro* assay (Fig. 3J); (ii) CESTA assays in treated cultured cells (Fig. 3H and I) and xenografts (Fig. 5G) provide evidence for target engagement; (iii) d16 induces cell-cycle arrest during the S-phase as expected from the outcome of DNA2 depletion (Fig. 3C and D); and (iv) DNA2 depletion decreases or mitigates the cytotoxic and anti-proliferative activities of d16 (Fig. 3E–G).

Treatment with d16 induces cell-cycle arrest mainly at S-phase and inhibits HR. These activities are consistent with the anticipated outcomes from DNA2 inhibition, regardless of the p53 status of the cancer cells. Thus, d16 not only inhibits proliferation of mutp53-transfected H1299 cells, but also p53-null H1299 cells and WT p53-transfected H1299 cells (Fig. 4A), although to a lesser degree.

### DNA2 as a Therapeutic Target in Mutant p53-harboring Cancers

Mutations in *TP53* are associated with therapy resistance and a poor prognosis in cancer. On the other hand, mutp53-bearing cancer cells appear to be more dependent on DNA2 for survival (9). This is likely attributed to the attenuation of ATR/Chk1 checkpoint response by mutp53, rendering cancer cells more vulnerable to DNA2 inhibition (9). Here we provide evidence for the employment of DNA2 inhibitors as the potential synthetic lethal therapy in mutp53-harboring cancers. Our data show that mutp53 expression renders cancer cells more sensitive to d16 (Fig. 4A); on the contrary, depletion of mutp53 decreases the sensitivity to d16 (Figs. 4B and 7D). Mechanistically, inhibition of DNA2 suppresses ATR/Chk1 checkpoint response to a greater extent in the mutp53-expressing cancer cells than in the p53-null cancer cells (Fig. 4C). Besides the inhibition of HU-induced Chk1 phosphorylation, d16 also increases the proportion of BrdU(+) cells without HU treatment (Supplementary Fig. S4B), consistent with the inhibition of ATR checkpoint. In addition to this underlying mechanism, it is clinically relevant that DNA2 overexpression can be driven by mutp53 (Fig. 1D and E) to gain survival advantage, and is frequently found in cancer, particularly in ovarian cancer (Supplementary Fig. S2). This makes DNA2 an attractive synthetic lethal therapeutic target in cancers with *TP53* mutations.

Data mining analysis assessing the clinical significance of DNA2 across TCGA human cancer types shows that DNA2 expression is upregulated in many types of cancer (Fig. 1A). *DNA2* gene is amplified in some tumors and contributes to the increase of DNA2 mRNA expression. However, the frequency of *DNA2* gene amplification is relatively low (0.33%, 33/9889 in TCGA Pan Cancer; 2.33%, 7/300 in TCGA ovarian cancer database). Consequently, the majority of DNA2 overexpression arises from alternative mechanisms. In particular, cancers or cancer cell lines harboring one of the hotspot mutp53 variants express higher levels of DNA2 than those harboring WT p53 (Fig. 1B–D). Interestingly, DNA2 protein levels are upregulated at an early stage of transformation in a broad range of cancer cells (20). However, the mechanisms underlying the link between mutp53 and DNA2 in cancer remain to be explored. By evaluating the clinical significance of DNA2 in patients with ovarian cancer, the cancer type with the most prevalent somatic *TP53* mutations, we found that high expression of DNA2 is associated with more advanced diseases, poor prognosis and acquired resistance to clinical response (Fig. 1F; Supplementary Fig. S2A–S2E). Interestingly, even within a more favorable ovarian cancer subpopulation, such as Stem-B, earlier stages or lower grades tumors, higher levels of DNA2 correlate to a worse patient outcome (Supplementary Fig. S2G, S2I, and S2K). Altogether, these data suggest that DNA2 can serve as a potential biomarker for more aggressive forms of cancers, particularly those expressing mutp53. It has been shown that mutp53 can promote the expression of many NF-Y target genes through its gain-of-function activity (6, 44). On the other hand, according to NFYA Gene Set in chromatin immunoprecipitation sequencing datasets from the ENCODE database, DNA2 can be a target of NF-Y (45). Thus, it seems plausible that mutp53 promotes the expression of DNA2 through its gain of function on NF-Y activity (6). DNA2 plays significant roles in cell-cycle regulation, DNA damage response, and HR-mediated DSB repair pathways (46). In most cancers, uncontrolled cell growth induces replication stress, resulting in the accumulation of DNA damages. As cancer cells can benefit from high levels of DNA2 as a defense mechanism, inhibitors that specifically target DNA2 can be developed to inhibit cancer cell proliferation.

### D16 Inhibits HR and Synergizes with PARPi in Cancer

We further exploit the synthetic lethality potential of DNA2 in ovarian and breast cancers by taking advantage of the role of DNA2 in HR repair. The clinical use of PARPis as a synthetic lethal therapy in BRCA1- and 2-deficient cancers has already been proved to be an effective strategy. Many studies demonstrated the crucial role of PARPs in DNA single-strand break (SSB) repair and at stalled replication forks (47–50). Inhibition of PARPs induces SSBs and DSBs accumulation in cells (51, 52). On the basis of these data, Liu and colleagues showed that PARP inhibition increased the effectiveness of DNA2 inhibitors (21). Herein, we demonstrated that d16 inhibits Rad51 recruitment and inhibits HR pathway (Fig. 6). d16 synergizes with various PARPis to reduce cell and tumor growth and induces apoptosis in ovarian and breast cancers. Furthermore, the combined treatment with PARPi and d16 shows a better effect in mutp53-expressing cells compared with mutp53-depleted cells (Fig. 7D), which is likely attributed to the effect of mutp53 on d16 response in these cells. Because the inhibition of HR by d16 is not dependent on mutp53, d16 and PARPi still show synergistic, albeit less effect in mutp53-depleted cells. The synergy between d16 and PARPi also supports the notion that DNA2-deficient or mutated tumors might be more sensitive to PARPi. Mutation of DNA2 can be seen in various types of cancer such as endometrial cancer (7.55%), stomach adenocarcinoma (3.18%) and bladder cancer (2.43%), etc. according to TCGA database.

In summary, DNA2 is highly expressed in many types of human cancer, and its expression is associated with mutp53 expression, disease stages, and poor outcomes. Through virtual docking and *in vitro* functional assay, we identify and validate d16 as a novel DNA2 inhibitor. Targeting DNA2 with d16 inhibits cancer cell survival and tumor growth *in vivo* in cancers harboring mutp53, and this effect is further enhanced when combined with other chemotherapeutic agents. Furthermore, as DNA2 plays a role in DSB repair via HR pathways, the combination of d16 and PARPis can be utilized as a synthetic lethal therapy in BRCA1- and or BRCA2-proficient cancer cells and xenografts. These findings provide opportunities to develop and characterize DNA2 inhibitors for synthetic lethality in mutant p53-bearing cancers.

## Supplementary Material

Supplementary Data Figures S1-S7, Table S1
