# Supplementary material for: DNA2 Nuclease Inhibition Confers Synthetic Lethality in Cancers with Mutant p53 and Synergizes with PARP Inhibitors
Source: Cancer Res Commun. 2023 Oct 16;3(10):2096–112. doi: 10.1158/2767-9764.CRC-23-0166 (PMC10578204; doi:10.1158/2767-9764.CRC-23-0166)
Supplement: Supplementary Data — Figures S1-S7, Table S1 [file crc-23-0166-s01.pdf]

Supplementary Data

Inhibiting DNA2 nuclease confers synthetic lethality to mutant p53-harboring cancers via DNA damage response and repair pathways.

Helena Folly-Kossi, Joshua D. Graves, Lidija A. Wilhelms Garan, Fang-Tsyr Lin and Weei-Chin Lin

Supplementary data contains:

Supplementary Fig. S1-S7

Supplementary Table S1

Supplementary References

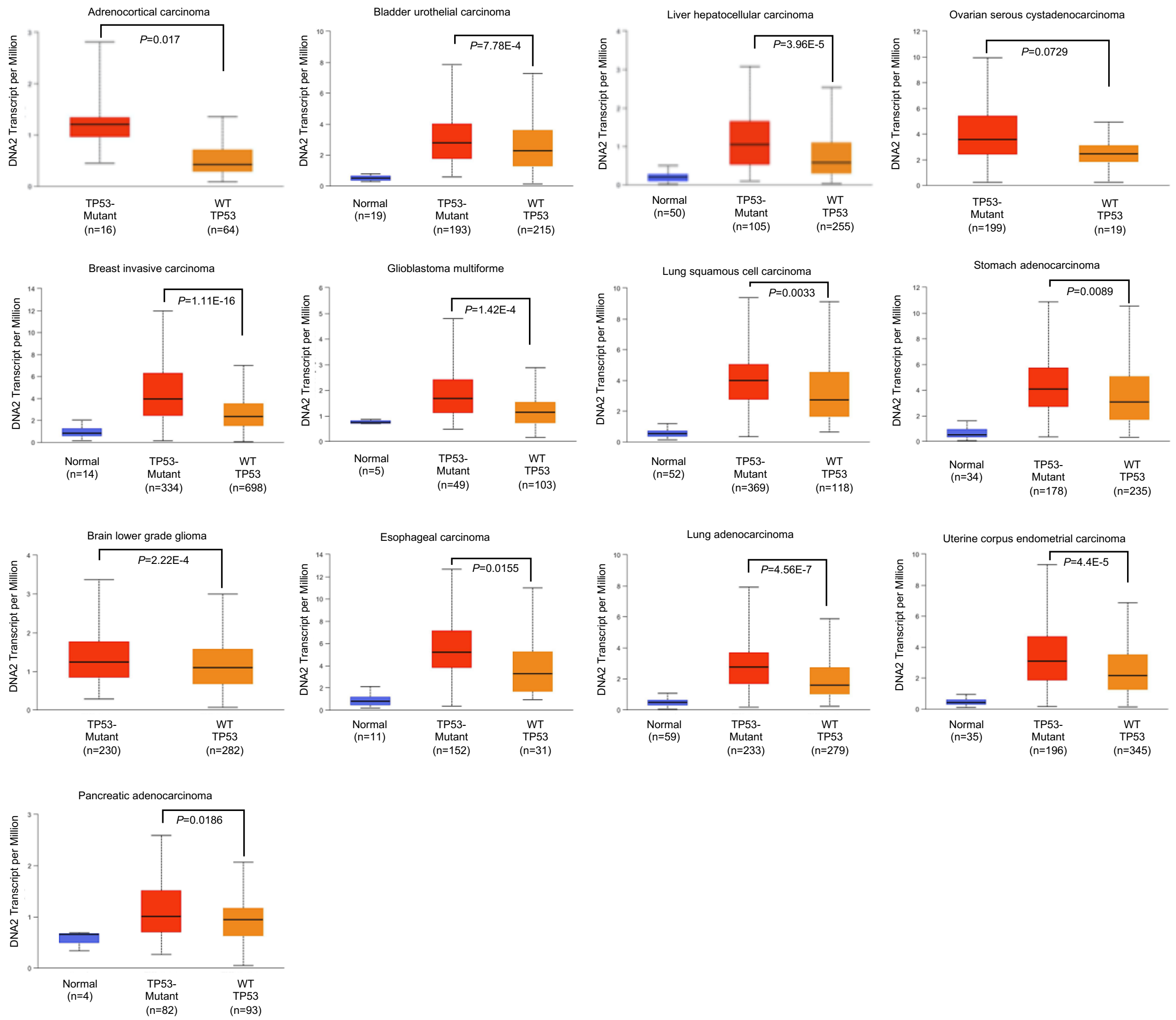

Supplementary Fig. S1.

Expression of DNA2 in normal tissues, *TP53*-mutated tumors, and *TP53* wild-type tumors in various types of cancer in TCGA datasets.

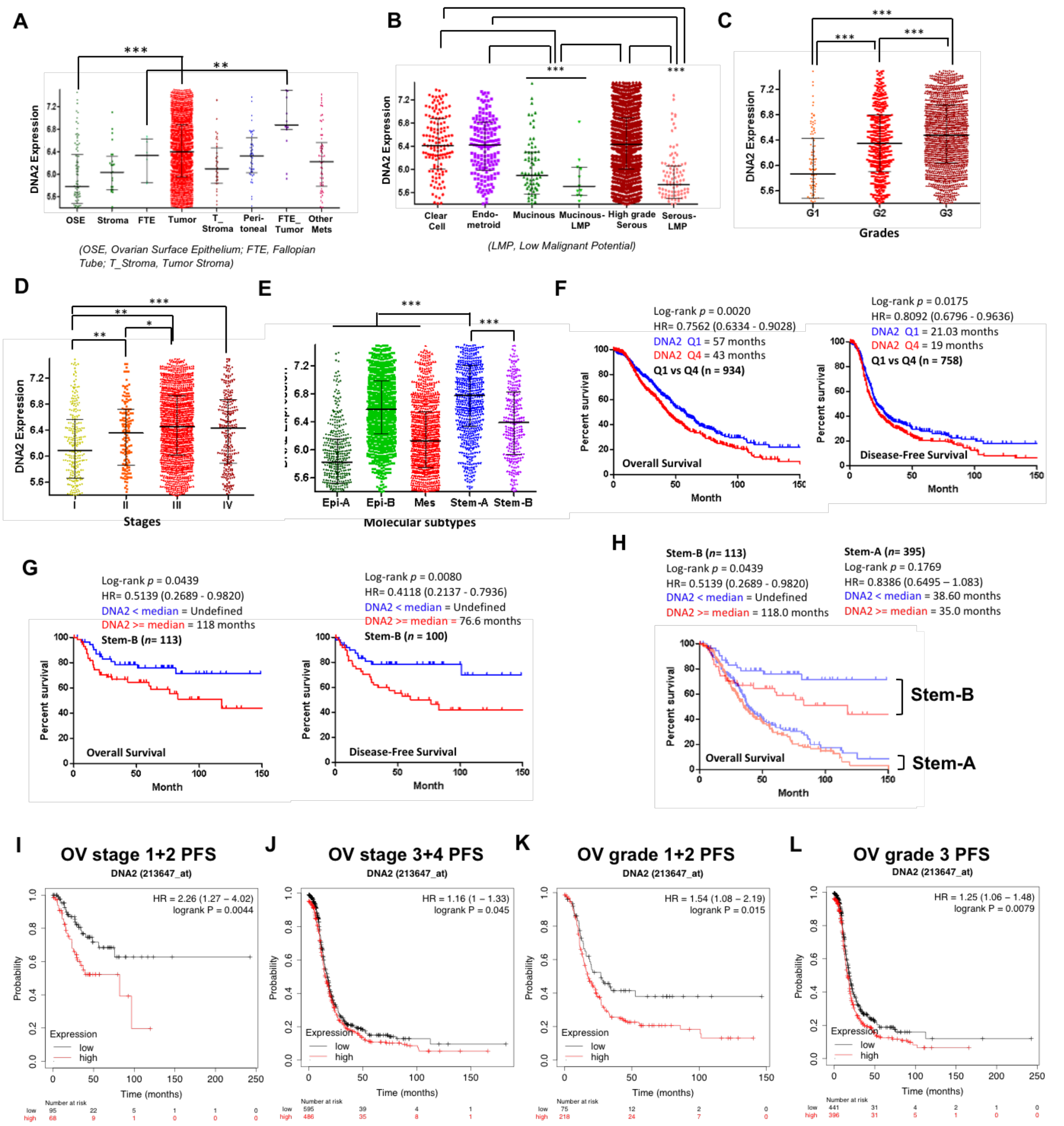

Supplementary Fig. S2. DNA2 overexpression is associated with poor clinical outcomes in epithelial ovarian cancer (EOC). (A-E) DNA2 expression profiles in (A) ovarian cancer status, (B) histological subtypes, (C) FIGO (International Federation of Obstetrics and Gynecology) grade, (D) FIGO stage and (E) molecular subtypes from CSIOVDB microarray gene expression database (1), where gene expression was normalized using frozen robust multiarray analysis

(fRMA) (2). (F-G) Kaplan-Meier survival curves showing the overall survival (left panel) and disease-free survival (right panel) based on the microarray gene expression database CSIOVDB in EOC (F) and in Stem-B (Stem-like B) molecular subtype (G). Q1: DNA2 expression in the lowest quarter of the whole cohort. Q4: DNA2 expression in the highest quarter of the whole cohort. Median expression was used in (G) to define DNA2 high levels in red text and DNA2 low levels in blue text. (H) Kaplan-Meier survival curves based on the microarray gene expression database CSIOVDB in Stem-B (Stem-like B) and Stem-A molecular subtypes. Median expression within each subtype was used to define DNA2 high levels in red text and DNA2 low levels in blue text. (I-L) Kaplan-Meier survival curves showing that high DNA2 expression is associated with shorter progression-free survival (PFS) in ovarian (OV) tumors, regardless of tumor stage or grade. DNA2 expression appears to have a better discriminating power in early stage or lower grade tumors. Data were analyzed in KM Plotter (3), which comprises 15 ovarian cancer datasets. HR: hazard ratio.

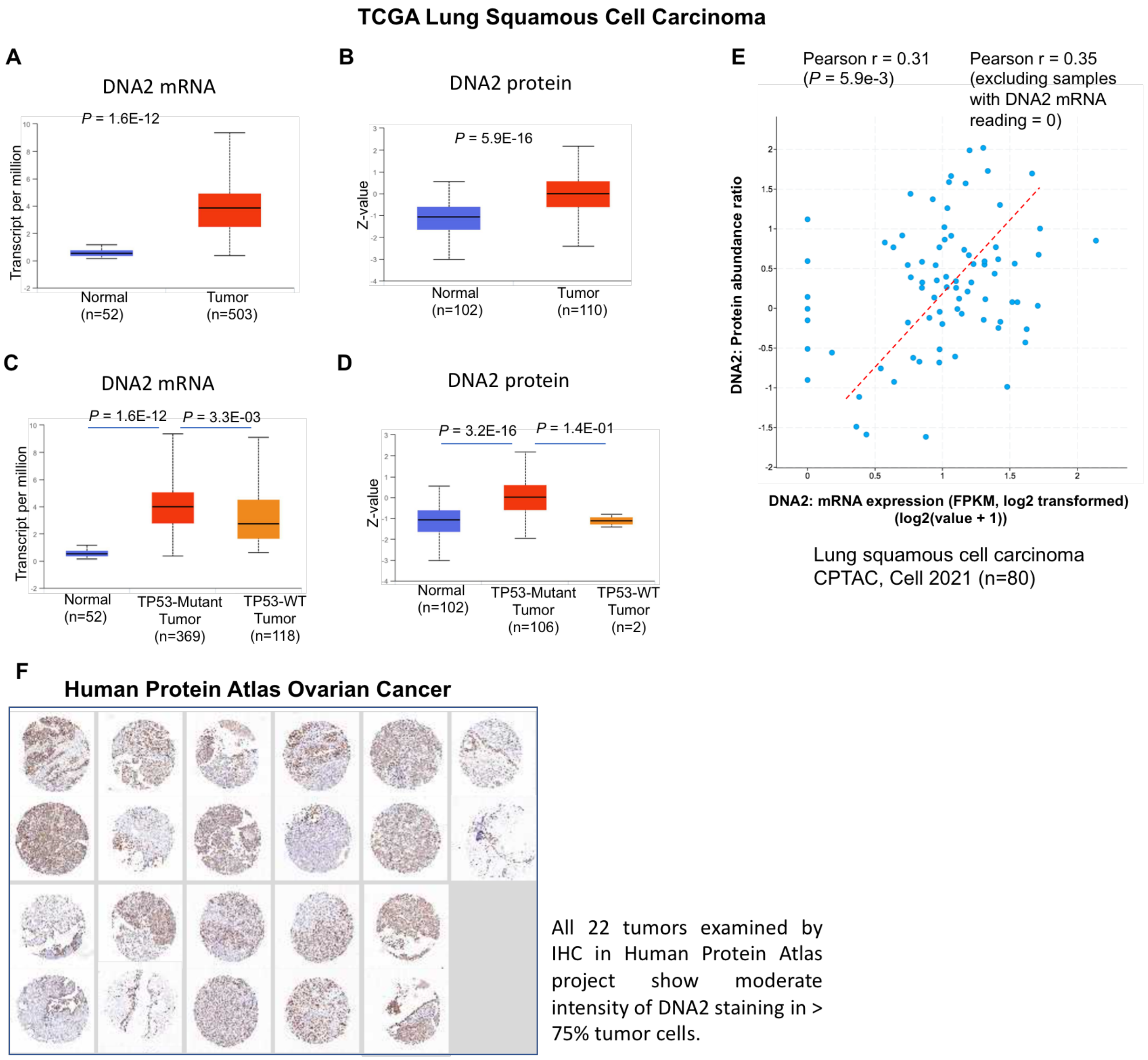

Supplementary Fig. S3. Overexpression of DNA2 protein in lung squamous cell carcinoma and ovarian cancer. (A-B) DNA2 mRNA and protein are overexpressed in lung squamous cell carcinoma as compared to their matched normal lung tissues in TCGA dataset. (C-D) DNA2 mRNA and protein are expressed at higher levels in mutant p53-bearing tumors compared with wild-type p53-bearing tumors. The comparison of proteins is not statistically significant since there are only two wild-type p53-bearing tumors in this cohort. (E) The expression of DNA2 protein is correlated with DNA2 mRNA in lung squamous cell carcinoma CPTAC dataset. (F)

All 22 ovarian cancers examined in Human Protein Atlas project (4) show moderate intensity of DNA2 protein signals in over 75% tumor cells.

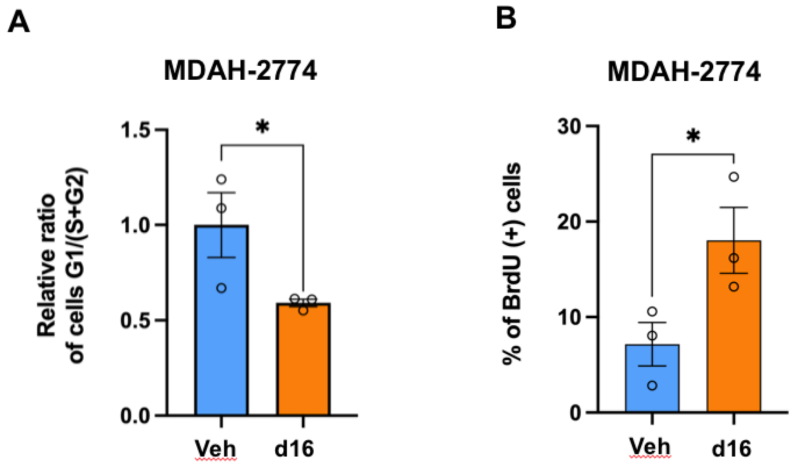

Supplementary Fig. S4. (A) Flow cytometric cell cycle analysis of MDAH-2774 cells treated with vehicle (Veh) or 20 μM d16 overnight. Shown is quantification of cell population in G1/(S+G2). Error bars = mean ± SEM. N = 3. *$P < 0.05$. **(B) Treatment with d16 in MDAH-2774 increases the population of BrdU(+) cells.** MDAH-2774 cells were treated with vehicle (veh) or 20 μM d16 overnight. Cells were then labeled with BrdU for one hour, followed by staining with a FITC-conjugated anti-BrdU antibody and analyzed by flow cytometry. Bar chart shows quantification of BrdU incorporation. Error bars = mean ± SEM. N = 3. *$P < 0.05$.

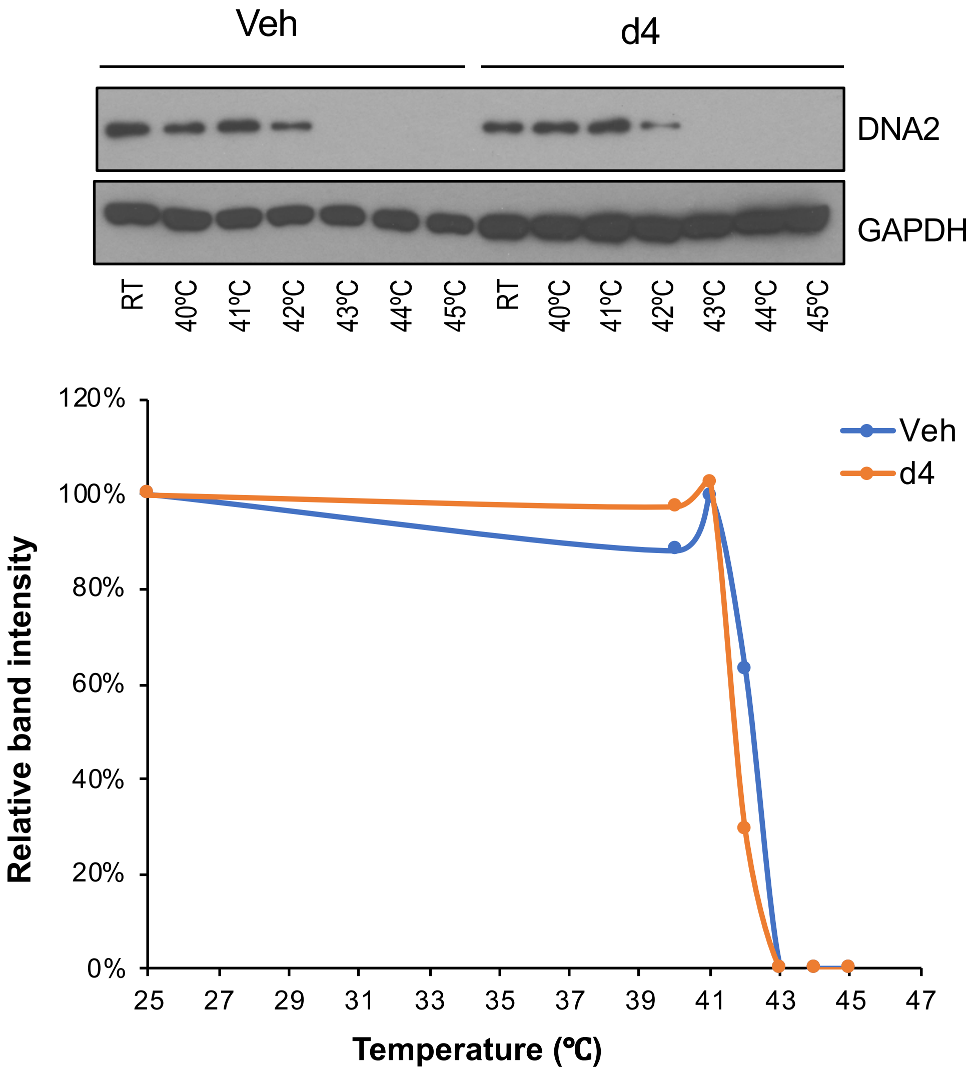

Supplementary Fig. S5. Cellular thermal shift assay (CETSA) of an inactive compound d4. Western blot and melt curves from MDAH-2774 cell lysates following d4 (20 μM) treatment. The intensity of DNA2 was quantified using NIH ImageJ software, normalized by its corresponding GAPDH signal, and relative to the room temperature (RT) sample.

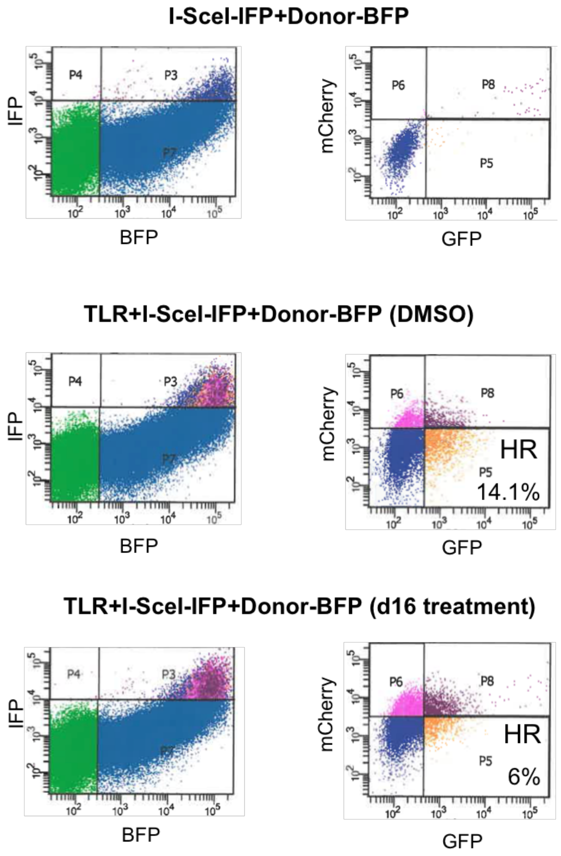

Supplementary Fig. S6.

Representative flow profiles of Traffic Light reporter assay in the result shown in **Fig. 6A**. Cells were first gated for the expression of both I-Sce (IFP) and donor constructs (BFP) (Left panels). The gated cells were then analyzed for GFP (HR) or mCherry (NHEJ).

A

Isobolograms (for Fig. 7B)

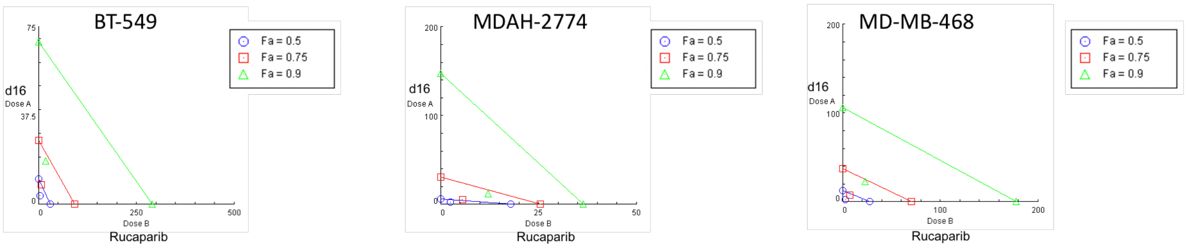

Combo	ED50	ED75	ED90	ED95
d16+RU	0.47025	0.39164	0.32854	0.29261

Combo	ED50	ED75	ED90	ED95
d16+Ru	0.54742	0.40272	0.41935	0.49799

Combo	ED50	ED75	ED90	ED95
d16+R	0.29881	0.32138	0.34686	0.36608

B **Fa-CI plots** (for Fig. 7D)

BT549 shScr vs shp53
d16+RUCA

C33A shScr vs shp53
d16+RUCA

MDAH2774 shScr vs shp53
d16+RUCA

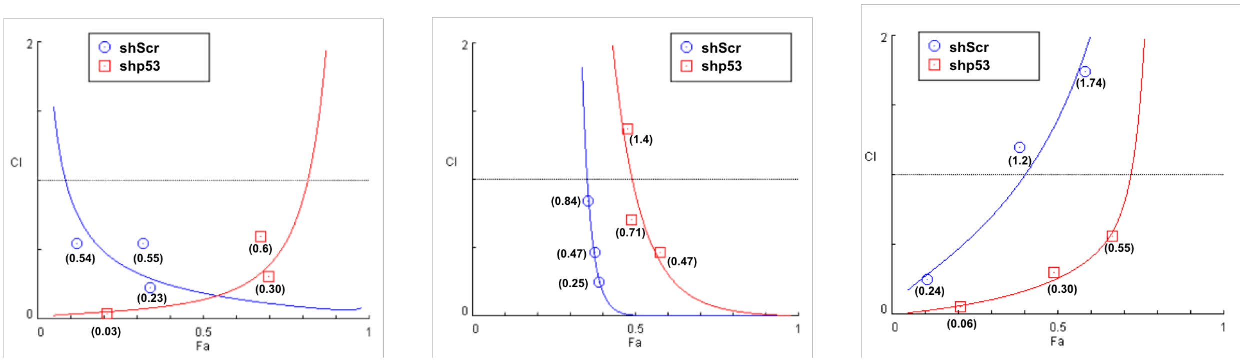

Supplementary Fig. S7.

(A) Isobolograms of Fig. 7B. **(B)** Fa-CI plots of Fig. 7D.

Supplementary Table 1

Compound ID in this study	MolPort ID	Molecular formula	Docking score
d1	MolPort-002-155-211	C15H15N3O5	-6.8
d2	MolPort-002-181-250	C16H13NO4	-6.7
d3	MolPort-006-192-374	C13H12FNO3	-6.5
d4	MolPort-000-651-064	C15H15NO5	-6.6
d5	MolPort-000-652-318	C14H13NO5	-6.1
d6	MolPort-022-868-828	C13H10FNO3	-6.7
d7	MolPort-000-651-077	C11H7NO5	-6.4
d8	MolPort-000-652-325	C13H13NO3	-6.5
d9	MolPort-000-876-343	C11H8ClNO3	-6.2
d10	MolPort-001-966-706	C11H8ClNO3	-6.6
d11	MolPort-002-563-934	C13H8F2N4O3	-6.9
d12	MolPort-007-988-595	C12H8F3NO3	-6.8
d13	MolPort-027-721-487	C21H17N3O7	-7.2
d14	MolPort-000-003-611	C12H9F2NO3	-6.5
d15	MolPort-000-085-382	C13H13NO3	-6.2
d16	MolPort-000-774-746	C16H11ClN2O6	-7.7
d17	MolPort-000-884-075	C12H10FNO3	-6.2
d18	MolPort-002-553-981	C18H20N4O5	-7.5
d19	MolPort-002-732-029	C12H10N2O5	-6.3
d20	MolPort-002-744-513	C11H8N2O5	-6.3
d21	MolPort-006-758-363	C13H12FNO3	-6.2
d22	MolPort-007-987-116	C11H8ClNO3	-6.5
d23	MolPort-007-987-120	C14H15NO3	-6.3
d24	MolPort-019-780-957	C19H13ClN2O5	-8.3

Supplementary Table 1.

A list of C5 analogs identified in our study with corresponding molecular formulas and virtual screening docking scores in DNA2.

Supplementary References:

1. Tan TZ, Yang H, Ye J, Low J, Choolani M, Tan DS*, et al.* CSIOVDB: a microarray gene expression database of epithelial ovarian cancer subtype. Oncotarget **2015**;6:43843-52
2. McCall MN, Bolstad BM, Irizarry RA. Frozen robust multiarray analysis (fRMA). Biostatistics **2010**;11:242-53
3. Gyorffy B. Discovery and ranking of the most robust prognostic biomarkers in serous ovarian cancer. Geroscience **2023**

4. Uhlen M, Fagerberg L, Hallstrom BM, Lindskog C, Oksvold P, Mardinoglu A*, et al.* Proteomics. Tissue-based map of the human proteome. Science **2015**;347:1260419
